# Hellenic karst waters: geogenic and anthropogenic processes affecting their geochemistry and quality

**DOI:** 10.1038/s41598-023-38349-6

**Published:** 2023-07-11

**Authors:** L. Li Vigni, K. Daskalopoulou, S. Calabrese, L. Brusca, S. Bellomo, C. Cardellini, K. Kyriakopoulos, F. Brugnone, F. Parello, W. D’Alessandro

**Affiliations:** 1 University of Palermo, DiSTeM, via Archirafi 36, Palermo, Italy; 2 Institute of Geosciences, University of Potsdam, Karl-Liebknecht-Str. 24-25, Potsdam-Golm, Germany; 3 German Research Centre for Geosciences, Wissenschaftpark “Albert Einstein”, Telegrafenberg, Potsdam, Germany; 4 Istituto Nazionale di Geofisica e Vulcanologia, Sezione di Palermo, via Ugo La Malfa 153, Palermo, Italy; 5 Dipartimento di Fisica e Geologia, University of Perugia, Via Pascoli Snc, 06123 Perugia, Italy; 6 Istituto Nazionale di Geofisica e Vulcanologia, sezione di Bologna, Viale Berti Pichat 6/2, 40127 Bologna, Italy; 7 Faculty of Geology and Geoenvironment, National and Kapodistrian University of Athens, Panepistimioupolis, Ano Ilissia, Athens, Greece

**Keywords:** Hydrology, Geochemistry, Environmental impact

## Abstract

Karst hydrosystems represent one of the largest global drinking water resources, but they are extremely vulnerable to pollution. Climate change, high population density, intensive industrial, and agricultural activities are the principal causes of deterioration, both in terms of quality and quantity, of these resources. Samples from 172 natural karst springs were collected in the whole territory of Greece. To identify any geogenic contamination and/or anthropogenic pollution, analyses of their chemical compositions, in terms of major ions and trace elements, were performed and compared to the EU limits for drinking water. Based on chloride content, the collected karst springs were divided into two groups: low-chloride (< 100 mg L^−1^) and high-chloride content (> 100 mg L^−1^). An additional group of springs with calcium-sulfate composition was recognised. Nitrate concentrations were always below the EU limit (50 mg L^−1^), although some springs presented elevated concentrations. High contents in terms of trace elements, such as B, Sr, As, and Pb, sometimes exceeding the limits, were rarely found. The Greek karst waters can still be considered a good quality resource both for human consumption and for agriculture. The main issues derive from seawater intrusion in the aquifers along the coasts. Moreover, the main anthropogenic pollutant is nitrate, found in higher concentrations mostly in the same coastal areas where human activities are concentrated. Finally, high levels of potentially harmful trace elements (e.g. As, Se) are very limited and of natural origin (geothermal activity, ore deposits, etc.).

## Introduction

Water resources are of critical importance for the development of life. Climate change has a severe impact on water availability, demand, and quality^1^, and it will result in the reduction of groundwater resources, particularly in the driest subtropical regions^2^. The stress on water resources is due to the increase in water demand for industrial and agricultural activities, and population growth, producing depletion both in quantity and quality of groundwater resources^3^. The report of IPCC^1^ estimates an increase in the world population under water scarcity between the twentieth and twenty-first centuries from 14 to 58%. Furthermore, climate change impacts also coastal groundwater; sea level rise, together with the over-pumping of coastal aquifers, produces dislocation of the saltwater-freshwater interface towards inland^2^.

Karst aquifers belong to the main freshwater resources. They occur in carbonate rocks (limestone and dolomite), which cover about 12% of the global land surface^4^. Thanks to the capacity of storing and transmitting huge amounts of good quality freshwater, karst hydrosystems are of worldwide economic interest, representing an important drinking water supply for about 25% of the world’s population^4^.

Karst hydrosystems are the result of intense water–rock interaction, known as karstification process, that involves the chemical dissolution of carbonate rocks (limestone, dolomite, and marble). The karstification phenomena occur also in evaporite formations (gypsum and anhydrite)^5^. Dissolution of carbonate rocks is favoured by carbon dioxide dissolved in water and occurs according to a set of equilibrium equations simplified as follows:

$${\text{H}}_{{2}} {\text{O }} + {\text{ CaCO}}_{{3}} + {\text{ CO}}_{{2}} \leftrightarrow {\text{ Ca}}^{{{2} + }} + {\text{ 2HCO}}_{{3}}^{ - }$$

The concentration of dissolved CO_2_ in meteoric recharge is controlled by temperature and partial pressure of CO_2_ in the atmosphere. Upon infiltration, carbon dioxide, that derives from biogeochemical processes within the soils (root respiration, microbial activity, biodegradation of organic matter, etc.) or from depth by geological processes, can complement the one coming from the atmosphere^6^.

Karst hydrosystems are particularly vulnerable to chemical and microbial contamination from several sources, climate change, and deterioration from overexploitation^5^, thus their protection and management are of critical importance. The main responsible for groundwater pollution-induced degradation, in the last decades, are agricultural, industrial, residential, and commercial activities, responsible for wastewater and fertilizer (nitrate) release, mining operations (heavy metals), seawater intrusion (chloride)^3,7,8^. The high vulnerability of karst aquifers is due to their peculiar hydrogeological and hydrodynamic features. Karst systems are characterized by strong hydraulic gradients, high flow velocities, flow rates, and short residence times^5^. Other important features are heterogeneity and anisotropy, which make it hard to develop a model of the systems to adapt their management. The heterogeneity in particular is due to a diversified network of high-permeability conduits and fractures where water follows preferential paths, which are directionally dependent on fractures geometry^3,4,9^.

Karst systems represent a strategic resource for the Hellenic territory. Indeed, in Greece, starting from the 1970s, interest in the research on karst systems considerably increased, because of the growing water demand. At first, the exploitation was limited to the discharge of springs, but the socioeconomic development of the country resulted in the expansion of population, agricultural and industrial activities and change of land uses within the karst aquifer boundaries^10,11^. One of the main sources of quality deterioration of groundwater resources comes from the intensive coastal exploitation, such as widespread urbanization and tourist development, causing aquifer salinization^12^. Agricultural activities represent the main use of water resources, with over 80% of total consumption^13^; therefore, other important sources of pollution in Greece are fertilizer use in agriculture, as well as disposal of untreated wastewater^12^.

Several studies were focused on the quality status of karst groundwaters in Greece, especially those used for irrigation and/or as drinking water resources. Most of them were focused on single karstic systems (e.g.,^14–16^), while others referred to wider areas^17,18^. Only few studies reported analyses of trace elements^19–23^. Further information about geology and hydrology of the Hellenic karst hydrosystems can be found in the supplementary material.

The karstic hydrologic systems of Greece represent a strategic water resource for the whole country that has to be protected both from the quantitative and qualitative point of view. The main aim of this work is to give an overview of the water quality in terms of the chemical composition of both major ions and trace elements, through the analysis of a large number of samples collected from the main karst springs of mainland Greece.

## Materials and methods

**Figure 1:**
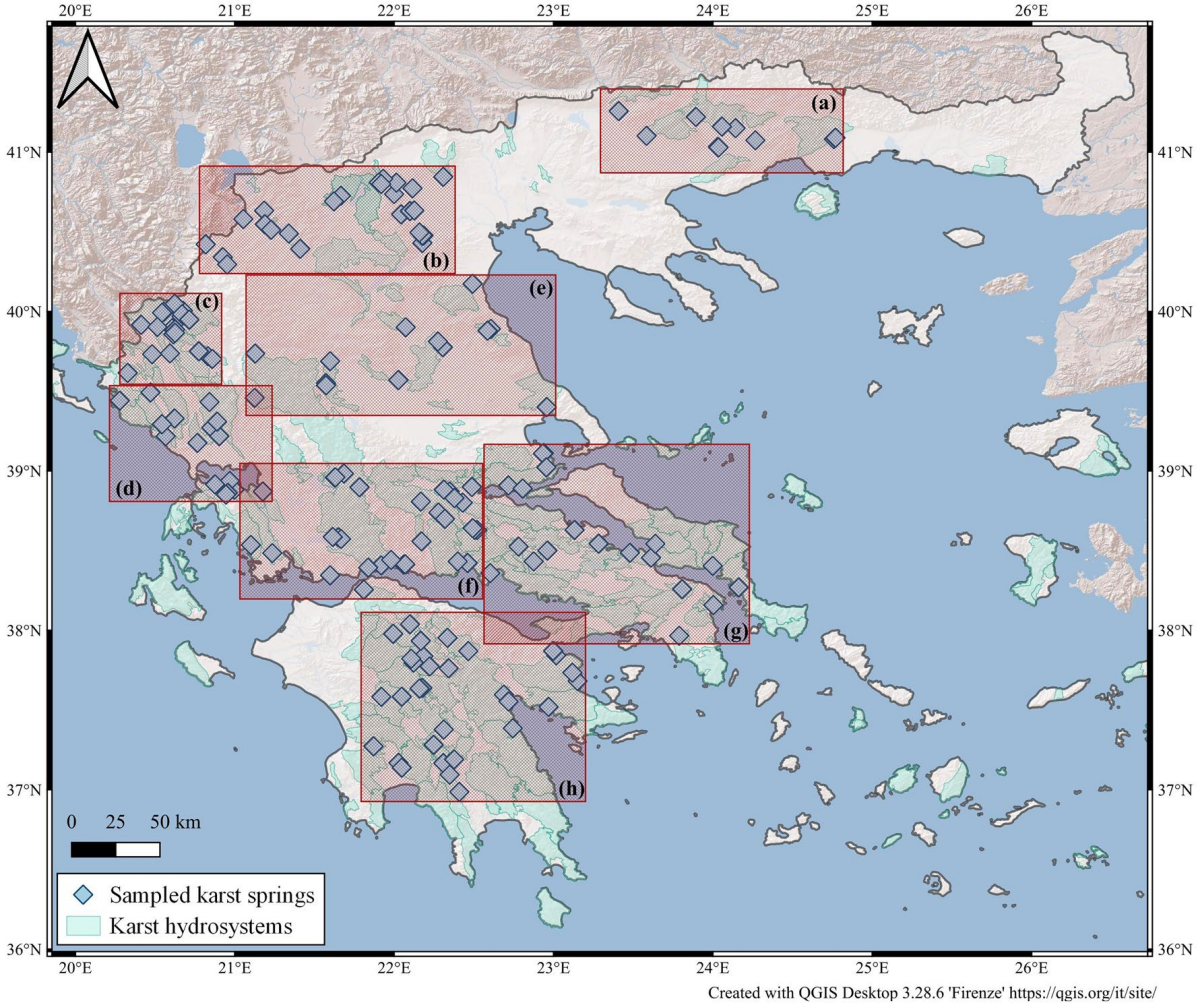
Geographic distribution of collected karst springs. Karst hydrosystems from^24^. The insets refer to Fig. SM1 (in supplementary material), where zoomed maps with the identification numbers of sampling sites are shown. Basemap by *ESRI* maps.

From May 2016 to October 2022, 172 karst water samples were collected along the Hellenic territory (Fig. 1) with their water chemistry being analysed at the laboratories of Istituto Nazionale di Geofisica e Vulcanologia (INGV-Palermo).

Samples were taken almost exclusively from natural springs. Only two samples (Mavrosoulava and Kaissarianis) were taken from drillings tapping karst aquifers. Sampling sites were selected mostly basing on the spring mean flow rates (> 50 L s^−1^). Only 13 samples were collected from springs with mean flow rate between 20 and 50 L s^−1^. Approximate position and flow rate data were taken prevailingly from the catalogue of Hellenic karst springs made by HSGME (Hellenic Survey of Geology and Mineral Exploration (former IGME) in the 1970s and 1980s^25–34^. Unfortunately, this catalogue does not cover the whole Greek territory, not comprising a few important areas (Attica, Epirus, Central Macedonia, Chalkidiki, and the Aegean islands). For these areas information was obtained from different publications (e.g.^20,35–39^). It is worth mentioning that mean flow rates may have changed significantly since the time that the original measurements took place. Indeed, three of the springs included in the catalogues with high measured flows at the time of the compilation, were at the time of our visit completely dry. Even though this work has covered the vast majority of karst springs with the highest water flow on the mainland, it should be noted that many big springs have not been sampled due to either imprecise geographic indications, or inaccessibility. Nevertheless, more than 80% of the catalogued springs with flow rates > 50 L s^−1^ have been analysed in the present study. Sampling date, geographical coordinates and mean flow rate are taken from literature^20,25–39^ of the collected springs and can be found in Table SM1.

Physico-chemical parameters, (water temperature, pH, redox potential (Eh), and electric conductivity (EC)) were measured in situ by portable instruments, whilst total alkalinity was determined by titration with 0.1 M HCl on unfiltered samples, expressed as mg(HCO_3_^−^) L^−1^. Water samples were filtered (0.45 µm MF-Millipore cellulose acetate filters) and stored in LDPE bottles for anion and isotope determinations, while an aliquot for the determination of cation contents was stored in Polypropylene (PP) bottles and acidified with ultrapure concentrated HNO_3_. Water chemistry was analysed using standard methods^40^. Major anions (F^−^, Cl^−^, NO_3_^−^ and SO_4_^2−^) and major cations (Na^+^, K^+^, Mg^2+^ and Ca^2+^) were determined by ionic chromatography (IC; Dionex ICS 1100). Silica (SiO_2_) was determined with Inductively Coupled Plasma Optical Emission Spectrometry (ICP-OES; Jobin Yvon Ultima 2).

For trace element analysis, filtered samples were stored in 50 ml PP bottles and then acidified to a pH of ~ 2 with ultrapure concentrated HNO_3_. Twenty-five trace elements (Li, Be, B, Al, Ti, V, Cr, Mn, Fe, Co, Ni, Cu, Zn, As, Se, Br, Rb, Sr, Mo, Cd, Cs, Ba, Tl, Pb, and U) were analysed by Inductively Coupled Plasma Mass Spectrometry (ICP-MS) using the following instruments: Agilent 7500ce for samples collected until 2018 and Agilent 7800 since then. Calibration solutions for all the investigated elements were prepared daily using an appropriate dilution of 100 mg L^−1^ and 1000 mg L^−1^ of stock standard solutions (Merck) with 0.14 mol L^−1^ ultrapure nitric acid. The accuracy of the method was checked by analysing certified reference materials of natural waters (Nist 1643e, Environment Canada TM-24.3 and TM-61.2, Spectrapure Standards SW1 and SW2) at regular intervals during sample analysis. The experimental concentrations determined in this study were in accordance with these certified values (within ± 10%).

Total dissolved solids (TDS) are here intended as the sum of all determined major ions plus silica. Speciation of solutions and saturation indexes of the relevant mineral phases for each sampled water were calculated using the aqueous speciation PHREEQC code^41^ with the thermodynamic data file *phreeqc.dat*.

Results were compared with the limits set by the Directive 98/83/EC^42^ and the Directive 2020/2184/EC^43^ that fix quality standards for waters suitable for human consumption.

## Results

### Major ions

Minimum, maximum and median values of the physico-chemical parameters as well as chemical compositions and saturation indexes of the main minerals of the collected karst water samples are reported in Table 1. The complete dataset can be downloaded from the Earthchem repository^44^.

**Table 1:** Statistical values of physico-chemical parameters, the chemical composition of major ions and trace elements, and of saturation index of the main minerals of the collected karst springs.

		Saline karst springs			Karst springs			Sulfate karst springs		
		Min	Max	Median	Min	Max	Median	Min	Max	Median
T	°C	11.3	33.5	17.3	5.6	25.0	13.9	11.3	29.0	13.1
pH		6.8	7.9	7.3	6.5	8.5	7.4	7.1	8.4	7.3
EC	μS cm^−1^	671	31,400	9775	175	1001	371	621	2700	1232
Eh	mV	90	175	127	35	399	167	− 38	193	136
Ca^2+^	mg L^−1^	53.7	367	205	24.5	291	71.0	147	599	305
Mg^2+^	mg L^−1^	3.94	975	256	0.595	43.1	7.66	18.4	57.4	28.3
Na^+^	mg L^−1^	99.5	7679	2035	0.805	37.0	4.21	4.30	301	7.25
K^+^	mg L^−1^	0.430	245	75.7	0.117	16.1	0.557	0.317	14.4	0.596
HCO_3_^−^	mg L^−1^	140	619	324	119	988	259	112	265	194
F^−^	mg L^−1^	0.015	1.11	0.100	0.015	0.382	0.086	0.091	0.973	0.443
Cl^−^	mg L^−1^	144	14,200	3639	0.120	81.1	5.56	3.24	547	7.44
NO_3_^−^	mg L^−1^	0.099	47.6	10.9	0.186	29.5	3.00	0.062	21.1	2.41
SO_4_^2−^	mg L^−1^	35.5	1936	550	1.54	98.5	7.10	278	1466	716
SiO_2_	mg L^−1^	2.78	48.5	18.7	3.24	19.4	8.07	0.302	21.6	8.65
Li	µg L^−1^	2.92	174	40.2	0.08	30.6	1.26	2.29	40.7	8.46
B	µg L^−1^	16.3	3871	883	1.00	198	8.96	18.1	124	46.2
Al	µg L^−1^	0.10	18.4	2.63	0.06	42.8	0.50	0.10	6.81	1.67
Ti	µg L^−1^	0.08	0.96	0.25	0.10	2.95	0.42	0.10	0.31	0.15
V	µg L^−1^	0.10	3.05	1.03	0.06	3.89	0.61	0.08	2.95	1.56
Cr	µg L^−1^	0.10	5.87	0.86	0.01	6.58	0.67	0.02	0.80	0.28
Mn	µg L^−1^	0.05	23.6	0.37	0.02	46.7	0.10	0.05	2.95	0.30
Fe	µg L^−1^	0.10	60.1	1.79	0.01	71.3	0.51	0.05	1.79	0.50
Co	µg L^−1^	0.01	0.87	0.02	0.01	0.40	0.04	0.01	0.07	0.02
Ni	µg L^−1^	0.02	6.91	0.54	0.02	3.84	0.14	0.05	2.77	0.28
Cu	µg L^−1^	0.02	1.95	0.10	0.02	11.5	0.10	0.05	0.22	0.10
Zn	µg L^−1^	0.05	40.8	0.22	0.05	68.1	0.40	0.05	0.73	0.18
As	µg L^−1^	0.06	12.1	2.12	0.01	17.0	0.25	0.04	1.18	0.57
Se	µg L^−1^	0.06	14.0	0.53	0.05	2.01	0.23	0.10	1.05	0.38
Br	µg L^−1^	21.5	52,717	7905	2.24	306	15.8	9.94	1519	15.5
Rb	µg L^−1^	0.61	98.6	19.7	0.09	54.7	0.40	0.26	4.34	0.55
Sr	µg L^−1^	261	7081	1884	41.1	1684	174	1053	8197	3417
Mo	µg L^−1^	0.23	12.0	1.53	0.01	2.93	0.39	0.19	10.9	3.64
Cd	µg L^−1^	0.01	0.99	0.02	0.01	0.05	0.01	0.01	0.05	0.04
Sb	µg L^−1^	0.01	0.44	0.08	0.01	0.37	0.03	0.02	0.40	0.14
Cs	µg L^−1^	0.03	6.89	0.34	0.00	3.77	0.01	0.01	0.36	0.03
Ba	µg L^−1^	16.2	111	28.1	3.67	179	19.9	10.8	28.8	17.2
Tl	µg L^−1^	0.01	0.16	0.03	0.00	0.20	0.01	0.00	0.08	0.01
Pb	µg L^−1^	0.01	2.36	0.02	0.01	1.00	0.01	0.01	0.82	0.02
U	µg L^−1^	0.12	3.11	0.95	0.02	2.99	0.32	0.13	1.48	0.76
Anhydrite	Saturation index	− 2.56	− 1.02	− 1.46	− 3.91	− 1.91	− 3.08	− 1.38	− 0.42	− 0.81
Gypsum		− 2.11	− 0.71	− 1.07	− 3.43	− 1.48	− 2.66	− 0.947	− 0.01	− 0.40
Halite		− 6.40	− 2.74	− 3.84	− 11.5	− 7.22	− 9.20	− 9.48	− 5.42	− 8.87
Aragonite		− 0.24	0.95	− 0.11	− 0.56	0.88	− 0.08	− 0.18	0.94	0.04
Calcite		− 0.09	1.09	0.04	− 0.40	1.03	0.07	− 0.04	1.09	0.19
Dolomite		− 0.97	2.78	0.47	− 1.96	0.93	− 0.61	− 0.70	1.40	− 0.32
Celestite		− 2.78	− 0.73	− 1.41	− 4.64	− 1.64	− 3.62	− 1.43	− 0.20	− 0.70
Strontianite		− 1.80	− 0.48	− 1.47	− 2.79	− 0.93	− 2.02	− 1.54	− 0.31	− 1.29
Fluorite		− 4.99	− 0.93	− 3.01	− 4.45	− 1.33	− 2.94	− 2.72	− 0.43	− 1.27

Temperature values range between 5.6 and 33.5 °C. The highest values were measured in Glyfa, Gouvo, and Kalamos Tsirloneri karst springs; sampling of these springs occurred during the summer period at the first accessible point, far from the main stream. Indeed, the strongest emission points in these springs are surrounded by reeds that make them inaccessible to sampling. For these springs the measured temperature was not considered representative of the groundwater conditions before emergence. The pH values vary from 6.5 to 8.5, whereas EC ranges from 174 to 31,400 μS cm^−1^ and Eh from − 38 to 399 mV.

**Figure 2:**
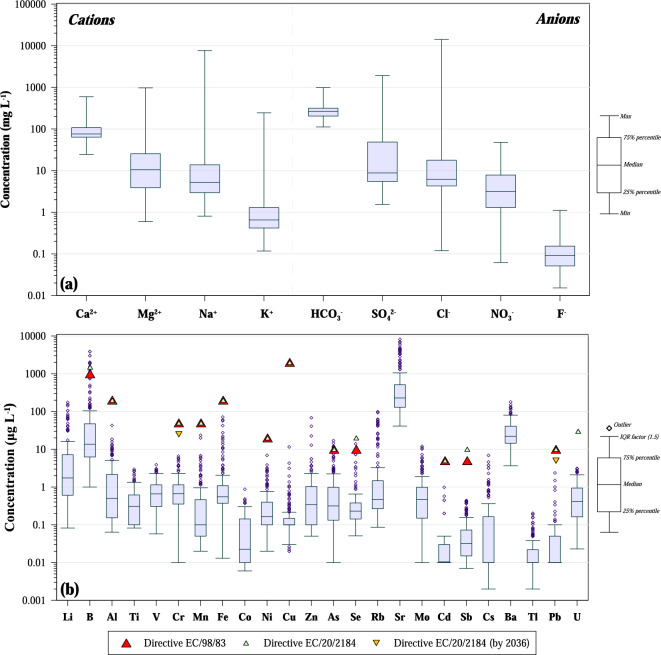
Boxplots of (**a**) major ions and (**b**) trace elements of collected karst springs.

The major ions show a large range of concentrations, sometimes four orders of magnitude. According to the median value, the concentration of, respectively, cations and anions decreases in the following order of abundance Ca^2+^  > Mg^2+^  > Na^+^  > K^+^ and HCO_3_^−^ > SO_4_^2−^ > Cl^−^ > NO_3_^−^ > F^−^ (Fig. 2a).

**Figure 3:**
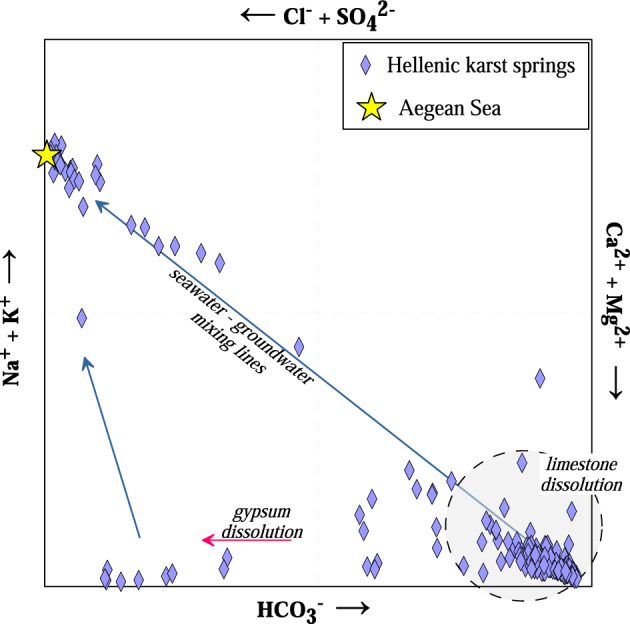
Langelier–Ludwig classification diagram^45^ where the Aegean Sea water end-member is signed as a star. The dashed circular area contains samples mainly affected by limestone dissolution. Red arrow indicates that gypsum dissolution process impacts some of the samples, while blue arrows represent seawater-groundwater mixing conditions.

All water samples were plotted in a Langelier–Ludwig diagram (Fig. 3), where three groups with different geochemical compositions can be recognised: (group a) characterized by Ca-HCO_3_ composition and low salinity (166–1343 mg L^−1^ of TDS); (group b) characterized by Na-Cl composition and high saline content (up to 25,653 mg L^−1^ of TDS); (group c) characterized by Ca-SO_4_ composition and low to medium salinity (674–2416 mg L^−1^ of TDS). The remaining samples have intermediate compositions often following mixing trends between the three groups.

### Trace elements

The collected karst waters were analysed also for trace elements determination (Table 1). They show a wide range of concentration, generally, more than two orders of magnitude (Fig. 2b). Trace elements can be subdivided into elements never exceeding 1 µg L^−1^ (Be, Co, Cd, Sb, Tl), 10 µg L^−1^ (Ti, V, Cr, Ni, Cs, Pb, U), 100 µg L^−1^ (Al, Mn, Fe, Cu, Zn, As, Se, Rb, Mo), 1000 µg L^−1^ (Li, Ba), and 10,000 µg L^−1^ (B, Sr). Sometimes the concentration was below the detection limit. Considering the concentration below the detection limit as a missing value, only four elements (Rb, Sr, Ba, U) show no missing data, whilst five elements (Mn, Cu, Cd, Tl, Pb) had less than 60% of determined values. Beryllium shows values always below the detection limit.

## Discussion

### Geogenic processes

To better discriminate saline and non-saline karst springs, samples were subdivided into two groups, according to low (< 100 mg L^−1^) and high (> 100 mg L^−1^) chloride content. Furthermore, based on their calcium-sulfate composition, an additional group, that includes 10 karst springs was recognised (Acheron, Bobos, Doliana, Gorgogouvli, Gouvo, Mana Nerou, Nelles, Rogozi, Vathy, and Zavarina Limni).

**Figure 4:**
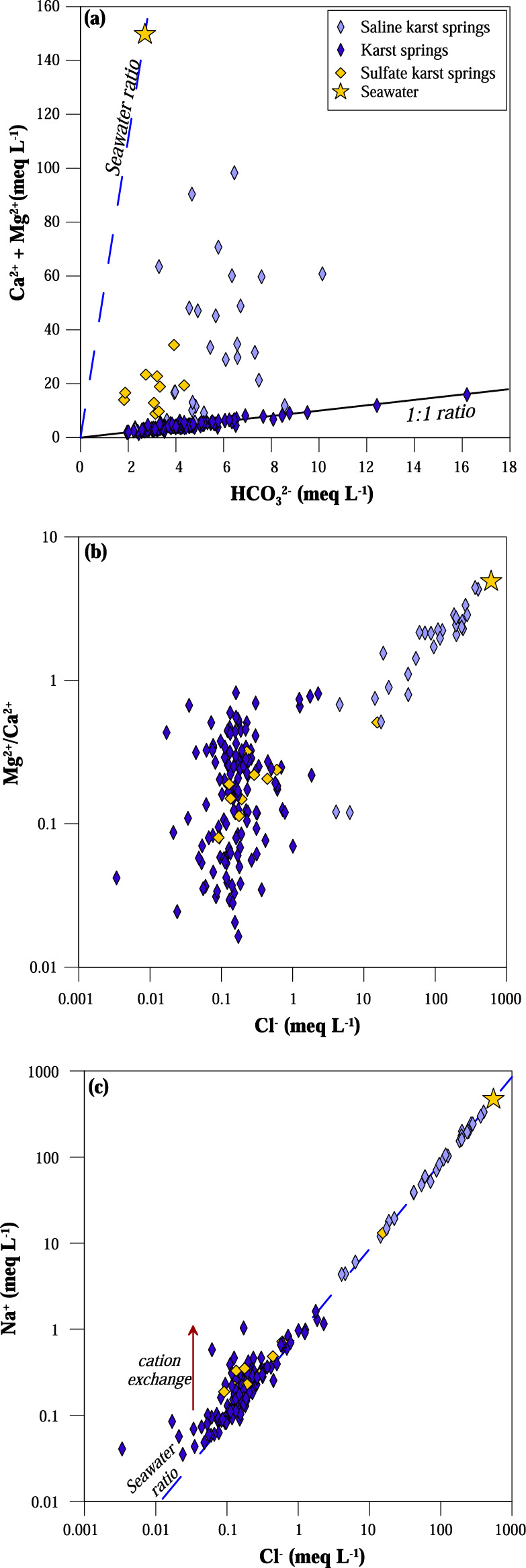
Binary correlation plots. (**a**) Ca^2+^ + Mg^2+^ versus HCO_3_^−^; (**b**) Mg^2+^/Ca^2+^ ratio versus Cl^−^; (**c**) Na^+^ versus Cl^−^. The Aegean Sea water point is also drawn in the plots. The Seawater ratio line is marked in plots (**a**) and (**c**), and the 1:1 equivalent ratio line is marked in (**a**). Red arrow represents cation exchange processes.

According to^24^, low chloride waters (group a) show the typical bicarbonate-alkaline-earth composition of groundwater circulating in carbonate aquifers. Carbonate dissolution process within the aquifers is confirmed by a good positive correlation between Ca^2+^ + Mg^2+^ and HCO_3_^−^ along the 1:1 equivalent ratio line (Fig. 4a). Saline and sulfate waters (groups b and c), instead, have an excess of Ca^2+^ and Mg^2+^ respect to the 1:1 equivalent ratio line; for the former, the excess can be explained with a seawater influence and is generally associated with a higher Mg^2+^/Ca^2+^ ratio tending towards that of seawater (Fig. 4b). The low salinity waters display on the same graph (Fig. 4b) a wide range in Mg^2+^/Ca^2+^ ratios not related to their Cl^−^ content. For these waters, the Mg^2+^/Ca^2+^ ratio depends on the mineralogy of the aquifers, with values tending towards a Mg^2+^/Ca^2+^ ratio of 1 when the prevailing rocks are dolomitic. On the contrary, low Mg^2+^/Ca^2+^ values are expected when calcite is the main constituent of the aquifers’ rocks. In the Na^+^ versus Cl^−^ binary diagram (Fig. 4c), the most saline waters fall along the seawater dilution line, confirming significant marine contamination of the aquifers. This is consistent with their coastal location. In the same diagram, some low saline waters show an excess of Na^+^ with respect to the seawater ratio line, suggesting that water–rock interactions within the aquifer may modify this ratio. The ionic exchange process between Ca^2+^ in water and Na^+^ in clay minerals may justify this pattern^46^.

**Figure 5:**
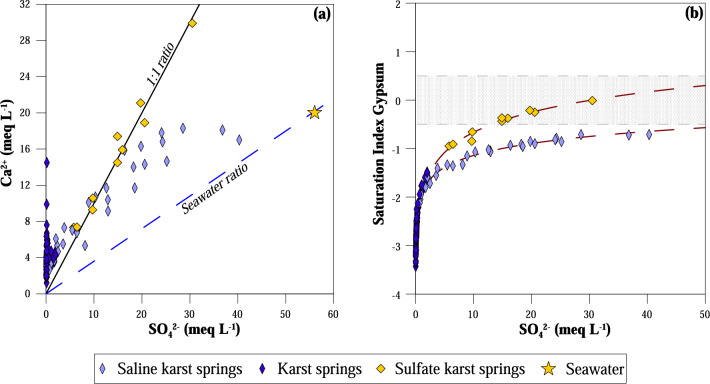
Binary correlation plots of sulfate karst springs. (**a**) Ca^2+^ versus SO_4_^2−^; (**b**) saturation index of gypsum versus SO_4_^2−^ (the shaded stripe is the ± 0.5 range of values in which the waters are considered at saturation with respect to the solid phase); (**c**) the geographical distribution of sulfate karst springs. Sea water ratio and 1:1 equivalent ratio lines are drawn in (**a**). Basemap by *ESRI* maps.

In a Ca^2+^ vs. SO_4_^2−^ binary diagram (Fig. 5a) group c samples plot along the 1:1 equivalent ratio line suggesting gypsum or anhydrite dissolution within their aquifer. To discriminate which sulfate-minerals have undergone dissolution, the saturation index of gypsum (Fig. 5b) and anhydrite were calculated; karst waters circulating in carbonate aquifers are all undersaturated both in gypsum and anhydrite, whilst the most sulfate-rich waters of group c reach saturation in gypsum but remain undersaturated in anhydrite (Table 1). The sulfate-composition of these waters is consistent with their geological environment. Springs with high sulfate content are located in Epirus (Fig. SM2). Their waters circulate within the Triassic pre-rift sequence of the Ionian zone^47^, which mainly consist of alternating gypsum formations and carbonate breccias cropping out close to major faults. According to previous studies^20,39^, the composition of sulfate springs is consistent with gypsum dissolution. On the other hand, for those samples collected near the shoreline, SO_4_^2−^ content mostly derives from seawater intrusion although some contribution from gypsum dissolution may in some cases not be excluded (Fig. 5a,b).

### Water quality

Water resources, in particular karst systems, are essential for the development of life. Thus, their management and protection are of crucial importance not only for human health but also for the correct balance of all terrestrial and marine ecosystems. Unfortunately, overexploitation and human activities (industry, agriculture, tourism) are the major responsible for the deterioration of water resources, in terms of quantity and, especially, quality.

Higher trace element concentrations are related to both natural and anthropogenic sources. In this respect, to recognize any anthropogenic impact on the aquifer systems, the knowledge of the hydrogeological setting together with the geological and structural features of the region is essential to discriminate the natural baseline^48^.

#### Salinity

The very great length of the Greek coastline, both absolute and relative to the extension of its territory, explains the high percentage of coastal karst aquifers feeding springs both at and under the sea level. The peculiarity of the Mediterranean area, which explains also the frequent occurrence of submarine karstic springs, derives from the important sea level drop that took place from 5.9 to 5.3 Ma before present during the Messinian Salinity Crisis^49^. If not isolated from the sea by impermeable sediments, these karst systems, which extend deep below sea level, may represent an easy inland access to contaminating seawater^50^. According to^24^, about 5% of the Greek karst hydrosystems is of poor quality due to seawater intrusion in coastal aquifers as a consequence of over-exploitation. Most of the karst springs sampled for this study have water of good quality for human consumption and are often used as drinking water. Only 15% of sampled springs have a high concentration of chloride and boron, often above the limits set by European Council for drinking water (Cl^−^ = 250 mg L^−1^; B = 1000 µg L^−1^), making them not suitable for human consumption. An example is the case of Almyri, Orea Eleni, and Selontas, in East Corinth, three springs of the same aquifer under strong anthropogenic stress; about twenty boreholes extract water from the aquifer in the upstream area, with a rate of 40–70 m^3^ h^−1^^51^, causing different degrees of salinization of the spring water, highlighted by the high chloride content (280–1250 mg L^−1^).

The distribution map of the sampled springs, which is subdivided in two classes (Cl^−^ above or below 100 mg L^−1^), is shown in Fig. SM4 (in supplementary material). All but one of the springs with high chloride content are located along the coast of Greece.

**Figure 6:**
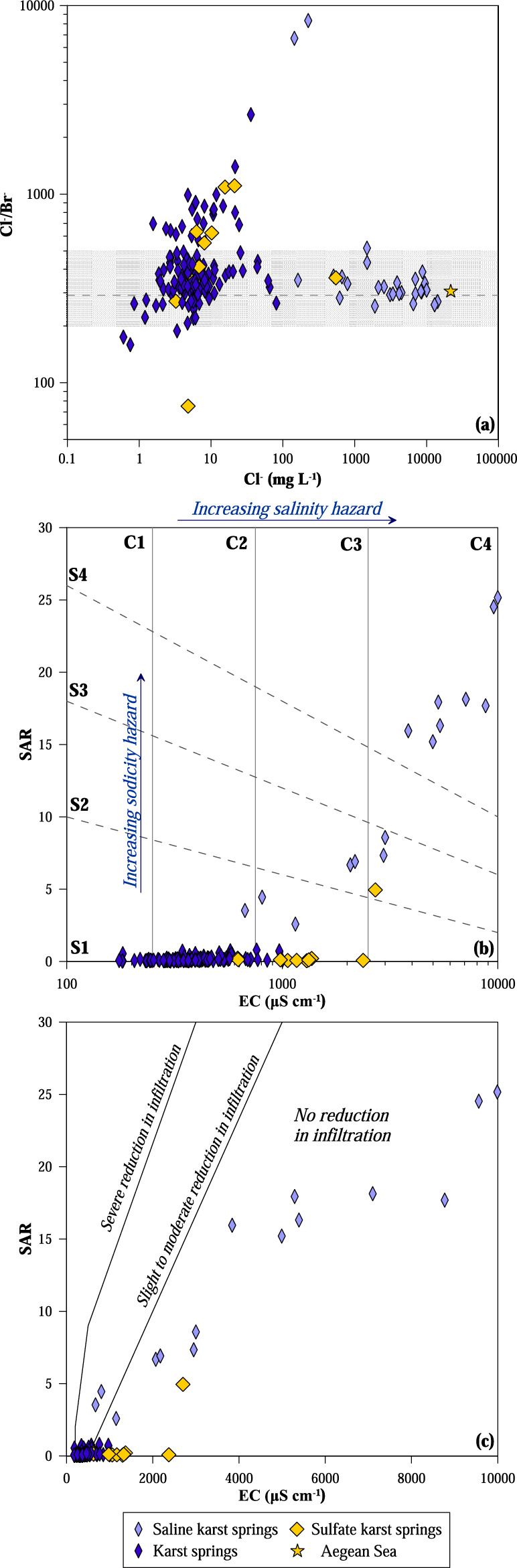
(**a**) Binary correlation plot of Cl^−^/Br^−^ ratio versus Cl^−^. The shaded stripe indicates the Cl^−^/Br^−^ ratios generally considered compatible with a marine source. Binary plots sodium adsorption ratio (SAR) versus electrical conductivity (EC), indicating plant toxicity classes (**b**) and soil permeability classes (**c**)^55^. Twelve samples with EC > 10,000 µS cm^−1^ fall outside the graphs. *C1* low, *C2* medium, *C3* high, *C4* very high salinity hazard, *S1* low, *S2* medium, *S3* high, *S4* very high sodicity hazard.

To better discriminate the possible sources of chloride, the Cl^−^/Br^−^ ratio was calculated (Fig. 6a). Most of the samples show a narrow range of values (200–500) similar to the seawater ratio (291—^52^). The most saline ones (Cl^−^ > 100 mg L^−1^), as evidenced before, denote a clear influence from seawater intrusion, while those with lower salinity reflect the Cl^−^/Br^−^ ratio of the meteoric recharge. Some low-salinity karst samples located in the Epirus area show higher chloride content than other nearby karst springs. Due to the great distance from the coast, the elevated chloride concentration cannot be related to present seawater intrusion. These samples show Cl^−^/Br^−^ ratio > 800 and belong to waters circulating within the Ionian zone sequences, where evaporite outcrops are present. In this respect, these Cl^−^/Br^−^ values may be ascribed to the dissolution of halite salt domes in the cores of anticlines^39^. Samples of Krya and Perama (IDs 100 and 101 in Fig. SM4), situated near Ioannina city, show the highest values of Cl^−^/Br^−^ ratio, 2642 and 8329 respectively: these values may be alternatively ascribed to the use of road salts (Cl^−^/Br^−^ > 5000^53,54^). Indeed, these springs are located in an area where winter temperatures often drop below zero and are very close to main roads where salts are used for de-icing purposes. Resampling of the spring of Perama at the end of the summer season revealed a similar high Cl^−^/Br^−^ ratio (6707) indicating that a natural origin from evaporite dissolution is the most probable explanation.

Comparatively, fewer water samples show Cl^−^/Br^−^ ratios below that of seawater. These are generally waters with low salinity (Cl^−^ generally below 10 mg L^−1^) and their Cl^−^/Br^−^ ratios may in some cases reflect lower values of their meteoric recharge. Alcalà and Custodio^56^ found that rainwater at high altitudes and/or inland areas are characterised by Cl^−^/Br^−^ ratios lower than that of seawater (down to < 100). Indeed, most of these samples were taken at high altitudes and far from the coast. This situation does not apply to the sample of Gorgogouvli (low altitude and close to the coast; ID 129 in Fig. SM4) where Br^−^ excess may be ascribed to anthropogenic sources, e.g., the use of agrochemicals^56^.

According to^13^, 80% of the total consumption of water resources in Greece is due to agricultural activities. Growth and yield of crops are strongly related to the quantity of dissolved salt in the soil waters. Although some plant species can grow in highly saline soils (e.g. halophytes), chloride, sodium, and boron have generally toxic effects on the growth of plants and may reduce the permeability of the soil^55^. In order to evaluate the salinity effects of soil water, salinity and sodium hazard index are used. The former considers the electrical conductivity of waters, indicating the value of 2250 µS cm^−1^ as the maximum salinity level in water for use in irrigation^55^. The potential sodium hazard is quantified using the sodium adsorption ratio (SAR), defined by the following equation:

1 $$\mathrm{SAR}=\frac{{\mathrm{Na}}^{+}}{\sqrt{{(\mathrm{Ca}}^{2+}+{\mathrm{Mg}}^{2+})/2}}$$

where the ion concentrations are expressed in milliequivalents per litre^57^.

The EC values of karst springs of this study range from 174 to 31,400 μS cm^−1^, with 20% of the samples, almost all belonging to the saline group, showing values falling in the high or very high salinity hazard fields. The highest EC values are associated with the karst springs located in coastal areas and affected by seawater intrusion into the aquifers. Moreover, nearly all of these waters show the highest SAR values, indicating an elevated potential for toxicity to plants (Fig. 6b). On the contrary, most of the saline waters do not create soil permeability problems due to the high sodium contents (Fig. 6c). Only the spring of Perama (Epirus), whose salinity is likely derived from evaporite dissolution, shows a moderate risk of soil permeability reduction (Fig. 6c). The low chloride karst springs group fall almost all in the low and medium salinity hazard classes (Fig. 6b) with a low sodicity hazard (< 2). Only few of the low-chloride karst springs fall in the high salinity hazard class, while almost all of the sulfate karst springs are included in this class (Fig. 6b); their salinity derives from the dissolution of evaporite rocks, mainly gypsum, but their sodicity hazard remains negligible. All these waters, falling in the high salinity hazard field, can still be used for irrigation provided that the irrigated soils are well drained preventing salt accumulation^55^. Moreover, the cultivation of salt-tolerant plant varieties allows often the use of waters belonging to the class of very high salinity hazard if no salt accumulation occurs in the soil. Especially in the areas of Greece characterised by a semi-arid climate, the use of salt-tolerant varieties has long been introduced, allowing the cultivation of vegetables sometimes with water conductivity up to nearly 10,000 µS cm^−1^^58^.

#### Nitrate

Nitrate is the most abundant nutrient, but it is considered also the most widespread pollutant. Although it may have a natural origin, such as atmospheric deposition or decay of organic matter, the main contribution derives from the increase of anthropogenic activities. The main anthropogenic sources are N-based fertilizers, untreated domestic and industrial wastewater, old septic systems, or leachate from landfill sites^3,59^. Nitrate is, often, added in excess to the soil to increase its productivity and most of it is leached to the aquifers below. Although there is no general consensus on the danger to human health represented by nitrate itself^60^, it remains an undesirable constituent generally accompanied by dangerous components (i.e., toxic agrochemicals or harmful microorganisms).

According to^12^, nitrate pollution is the second major source of groundwater degradation in Greece Many aquifers in Greece display high nitrate content, exceeding the European maximum admissible concentration (50 mg L^−1^) for drinking water^12,61^, making them non-suitable for human consumption. The most affected aquifers, with values exceeding the European limits, are the Boeoticos Cephissos hydrosystems in Central Greece^17^, the Vocha plain in Korinthos prefecture^62^, Thessaly district^12^. The main source of nitrate is the excessive application of fertilizers (NH_4_NO_3_, (NH_4_)_2_SO_4_, and nitrogen phosphate potassium) in intensively cultivated lands (such as for cotton, tobacco, and olive). Other sources of nitrate are septic tanks and untreated domestic effluent from abandoned wells in urban areas^62^.

**Figure 7:**
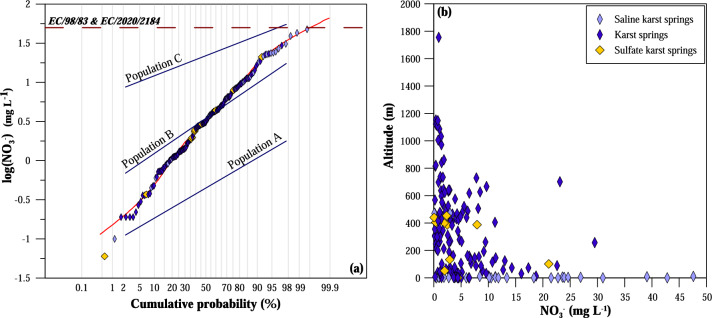
(**a**) Probability plot of NO_3_^−^ concentration. The red dashed line represents the NO_3_^−^ limit fixed by the European Directives (50 mg L^−1^) The black dashed lines represent the three partitioned populations and the three lines the theoretical statistical distribution resulting from the combination of 12% population A, 71% population B and 17% population C; (**b**) binary correlation plot of altitude versus NO_3_^−^.

In this study, nitrate concentration in karst springs never exceeds the European limit for drinking water (50 mg L^−1^^43^), suggesting a good quality for the majority of the sampled karst groundwater. Nevertheless, high NO_3_^−^ content was found in some springs, with values up to 47.6 mg L^−1^ (Fig. 7a). Three main log-normal populations were recognised from the probability plot using the partition procedure proposed by^63^ (Fig. 7a). Population A is characterised by an average value of ~ 0.6 mg L^−1^ and is mainly represented by waters with the lowest NO_3_^−^ concentrations (< 1 mg L^−1^). These populations can be considered representative of un-polluted water, representing the natural background conditions. Population B is characterised by a mean NO_3_^−^ of ~ 4.8 mg L^−1^, suggesting only limited input from anthropogenic sources. The third population (C) has the highest average NO_3_^−^, ~ 24.5 mg L^−1^, and a 95th percentile of ~ 47 mg L^−1^. The most representative springs of this population (evidenced in red in Fig. SM5) are located close to urban centres, farmland, or highly touristic coastal areas, clearly evidencing pollution issues. The distribution map of nitrate concentrations (Fig. SM5) can be compared to the maps of the main agricultural areas and the population densities in Fig. SM6 (supplementary material).

In a correlation with the altitude (Fig. 7b), the lowest nitrate concentrations were found in mountain areas, whilst the most polluted springs are located in coastal areas, which are heavily exploited for agriculture and tourism and where urban centres are widely present. Exceptionally, some springs (Petres, Kefalovryso Karpenisi, Santovou, Xino Nero, and Tria Piagadia—IDs 11, 33, 125, 146 and 171 in Fig. SM5), although located at about 600–800 m of altitude, display an elevated nitrate content (up to 23 mg L^−1^). These derive from higher altitude, urbanised, and intensively cultivated intramountain basins.

#### Arsenic

Arsenic is considered a highly toxic metalloid, which has harmful effects on human health, classified as a class1 carcinogen by the International Agency for Research on Cancer^64^. The European Council has established the value of 10 µg L^−1^ as the maximum contaminant level for drinking water. Arsenic contamination may have both natural and anthropogenic origins: it can be naturally derived from the chemical weathering of sulfide ore deposits or transported by geothermal waters, whilst the main anthropogenic sources are mining activity, coal combustion, and As-pesticides^65,66^.

Many regions of Greece, especially in the northern part, are affected by elevated concentrations in groundwater^67^. The highest As concentration was found in the groundwater of the geothermal area of Chalkidiki in northern Greece, with values up to 1000 µg L^−1^^68^. The main sources of As in Greece are geothermal fluids arising from active tectonic and volcanic areas^67^.

Data on arsenic in Hellenic karst water are very scarce^19,21–23^. However, in the present study values exceeding the European limit were found in three karst springs (Fig. 2b): Tempi (ID 39; up to 17.0 µg L^−1^), Potamos (ID 63; 12.1 µg L^−1^), and Paleomylos (ID 51; 12.0 µg L^−1^). The three springs (shown in red in Fig. SM7 with their IDs) are found in the eastern part of Thessaly and Central Greece and the arsenic contamination can be related to the geological settings. Indeed, the involved karstic systems were formed within carbonate formations at the contact with metamorphic and metavolcanic formations of the Ampelakia Unit (Blueschist unit) and Pelagonian Unit^15^. Many occurrences of As-rich mineralisations have been found in the area mainly related to the metamorphic rocks^67,69^. In some cases, the As contamination can be related to the presence within the aquifer of As-rich Karst-Type Bauxites^70^. For comparison, the distribution of the main industrial areas and the main mineralizations in Greece are shown in Fig. SM8 (supplementary material).

Although not showing extreme As concentrations like that of many geothermal waters in Greece^71^, the impact of these waters should not be disregarded. Because these As-rich karstic waters were sampled from springs with large flows (up to more than 2000 L s^−1^), even concentrations not strongly exceeding the maximum allowed level correspond to large As fluxes that may have an adverse influence on the ecosystems fed by these waters.

Further discussion about other trace elements (Sr, Cr, Ni and Pb) in the karstic waters of Greece can be found in the supplementary material.

## Conclusion

The main hydrogeochemical types of karst water in Greece are calcium-bicarbonate for hinterland springs and sodium-chloride for coastal karst aquifers. Furthermore, a third hydrogeochemical group of waters whose calcium-sulfate composition derives from the dissolution of gypsum within their aquifers has been recognised. Trace elements contents are generally low except for elements associated with carbonate or sulfate minerals dissolution (B, Sr and Ba). Drinking water limits are rarely exceeded except for parameters related to seawater contamination in the coastal aquifers (EC, Na, Cl, B). In these areas most of the human population and activities are concentrated and, therefore, also the highest nitrate levels are found, though always below the drinking water limit. Among the remaining elements only As and Se exceed in few cases their maximum admitted contaminant limits. Such exceedance could not be related to anthropogenic activities and probably derives from present or past hydrothermal activities.

Excluding those waters with EC > 10 mS cm^−1^, most of the waters unsuitable for drinking purposes due to high salinity may still be used in agriculture to irrigate salinity and boron-tolerant plant varieties on well-drained soils that do not allow salt accumulation.

The present study, while not covering the totality of the Greek big karstic springs, represents a first attempt to give a homogeneous dataset on the geochemistry of the waters circulating in the karst hydrosystems of the country. This dataset gives precious information about the quality status of these waters, even though it considers only the main ionic species and a large set of trace elements. On this basis, further studies should investigate also possible microbiological contaminations, the presence of organic pollutants or other potentially harmful trace elements (e.g. technological critical elements) and should also define the origin of the few trace element contaminations found in this study. Furthermore, among these springs, those representing the most important water resources should be chosen to follow up in time the most important quality indexes, in order to correctly manage this precious asset.

Notwithstanding the above limitations, this study shows that at present the Greek karstic hydrosystems, at least those located far from the coast, have to be considered a still intact water resource of national interest. These systems, being generally prone to contaminant infiltration, have to be carefully protected. Most of the population and human activities are concentrated in coastal areas where the karstic aquifers are often naturally contaminated by seawater intrusion, although sometimes salinity is increased by overpumping. In recent times human activities, that have the potential to contaminate precious water resources, are extending also towards mountainous areas. Luckily many recharge areas of important karstic aquifers are included within natural reserve areas, but it is of utmost importance to preserve also those outside these areas. In order to succeed, different administrative policy is applied in the numerous hydrologic basins of Greece^72^, where strategic (principles and planning) and functional (implementation of measures and actions until the final user) management of the water resources takes place^73^.

## Supplementary Information

Supplementary Information.

## Data Availability

The datasets generated during the current study can be obtained from the Earthchem Repository^44^.
