# Supplementary material for: Hellenic karst waters: geogenic and anthropogenic processes affecting their geochemistry and quality
Source: Sci Rep. 2023 Jul 11;13:11191. doi: 10.1038/s41598-023-38349-6 (PMC10336041; doi:10.1038/s41598-023-38349-6)
Supplement: Supplementary file 1 — Supplementary Information. [file 41598_2023_38349_MOESM1_ESM.docx]

**Hellenic karst waters: geogenic and anthropogenic processes affecting their geochemistry and quality**

Li Vigni L.^1^, Daskalopoulou K.^2-3^, Calabrese S.^1-4^, Brusca L.^4^, Bellomo S.^4^, Cardellini C.^5-6^, Kyriakopoulos K.^7^, Brugnone F.^1^, Parello F.^1^, D’Alessandro W.^4*^

1) University of Palermo, DiSTeM, via Archirafi 36, Palermo, Italy

2) University of Potsdam, Institute of Geosciences, Karl-Liebknecht-Str. 24-25, Potsdam-Golm, Germany.

3) German Research Centre for Geosciences, Wissenschaftpark "Albert Einstein", Telegrafenberg, Potsdam, Germany

4) Istituto Nazionale di Geofisica e Vulcanologia, sezione di Palermo, via Ugo La Malfa 153, Italy

5) University of Perugia, Dipartimento di Fisica e Geologia, Via Pascoli snc, 06123 Perugia, Italy

6) Istituto Nazionale di Geofisica e Vulcanologia, sezione di Bologna, Viale Berti Pichat 6/2, 40127 Bologna

7) National and Kapodistrian University of Athens, Faculty of Geology and Geoenvironment, Panepistimioupolis, Ano Ilissia, Greece

corresponding author: [walter.dalessandro@ingv.it](mailto:walter.dalessandro@ingv.it)

**Supplementary Information**

**Table SM1**. Identification number, geographical coordinates and mean flow rate taken from literature of the collected Hellenic karst springs.

| **ID** | **Name** | **Date** | **Lat** | **Long** | **Flow Rate** (L s^-1^) |
| --- | --- | --- | --- | --- | --- |
| 1 | Stratones | 18/05/2016 | 41.088814 | 24.767511 | 683 |
| 2 | Agios Ioannis Paradisos | 18/05/2016 | 41.081509 | 24.758688 | 533 |
| 3 | Kefalari | 19/05/2016 | 41.073990 | 24.265921 | 1000 |
| 4 | Drama | 19/05/2016 | 41.150394 | 24.141744 | 2778 |
| 5 | Milopotamos | 19/05/2016 | 41.159827 | 24.056483 | 556 |
| 6 | Aggitis | 19/05/2016 | 41.220667 | 23.893010 | 2778 |
| 7 | Symboli | 19/05/2016 | 41.028870 | 24.035283 | 278 |
| 8 | Ano Symboli | 19/05/2016 | 41.032879 | 24.030773 | 1000 |
| 9 | Eptamyli | 20/05/2016 | 41.102047 | 23.583489 | 278 |
| 10 | Sidirokastro | 20/05/2016 | 41.257393 | 23.408329 | 278 |
| 11 | Petres | 21/05/2016 | 40.726037 | 21.665075 | 111 |
| 12 | Stratopedo (Xanthi) | 22/03/2017 | 41.090362 | 24.769230 | 56 |
| 13 | Mexiates | 11/05/2017 | 38.882183 | 22.310921 | 300 |
| 14 | Kombotades | 11/05/2017 | 38.870196 | 22.347968 | 81 |
| 15 | Mavroneri | 11/05/2017 | 38.800668 | 22.429668 | 125 |
| 16 | Agia Anna | 12/05/2017 | 39.775873 | 22.304607 | 153 |
| 17 | Mati | 12/05/2017 | 39.811902 | 22.273927 | 1631 |
| 18 | Kefalokryso | 12/05/2017 | 39.900731 | 22.071745 | 1184 |
| 19 | Voda | 15/05/2017 | 40.809219 | 21.902661 | 1000 |
| 20 | Nisia | 15/05/2017 | 40.832149 | 21.930348 | 333 |
| 21 | Karkagia | 15/05/2017 | 40.808249 | 22.013392 | 1194 |
| 22 | Flamouria | 15/05/2017 | 40.736899 | 21.999048 | 69 |
| 23 | Sevastiana | 15/05/2017 | 40.772960 | 22.114301 | 403 |
| 24 | Aristotelis | 15/05/2017 | 40.630052 | 22.098653 | 528 |
| 25 | Agios Nikolaos | 15/05/2017 | 40.609505 | 22.043057 | 4722 |
| 26 | Kopano | 15/05/2017 | 40.633911 | 22.125795 | 153 |
| 27 | Artemis | 16/05/2017 | 40.429848 | 22.176380 | 20 |
| 28 | Georgianos | 16/05/2017 | 40.474526 | 22.176208 | 69 |
| 29 | Stratopedo | 16/05/2017 | 40.477934 | 22.177592 | 389 |
| 30 | Rachia | 16/05/2017 | 40.476888 | 22.184236 | 806 |
| 31 | Tripotamos | 16/05/2017 | 40.495811 | 22.158138 | 1000 |
| 32 | Aravissos | 14/10/2018 | 40.844870 | 22.307440 | 694 |
| 33 | Santovou | 15/10/2018 | 40.393070 | 21.409720 | 76 |
| 34 | Istekou | 15/10/2018 | 40.489110 | 21.338490 | 148 |
| 35 | Lefki | 15/10/2018 | 40.512510 | 21.226930 | 572 |
| 36 | Koromilia | 15/10/2018 | 40.547160 | 21.187880 | 444 |
| 37 | Gavros | 16/10/2018 | 40.632480 | 21.183530 | 146 |
| 38 | Ieropigi | 16/10/2018 | 40.579980 | 21.052480 | 167 |
| 39 | Tempi | 16/10/2018 | 39.879400 | 22.585270 | 772 |
| 40 | Anavras est | 17/10/2018 | 39.569500 | 22.025560 | 64 |
| 41 | Voula | 17/10/2018 | 39.569424 | 22.025554 | 217 |
| 42 | Gkouras | 17/10/2018 | 39.687778 | 21.597500 | 111 |
| 43 | Gorgogyrio | 17/10/2018 | 39.550278 | 21.571667 | 583 |
| 44 | Xilopariko | 17/10/2018 | 39.536667 | 21.571667 | 265 |
| 45 | Kaliakuda | 17/10/2018 | 39.400020 | 22.956808 | 85 |
| 46 | Livari | 17/10/2018 | 39.111444 | 22.938168 | 20 |
| 47 | Nerotrovias | 18/10/2018 | 38.829722 | 22.377500 | 69 |
| 48 | Mantania | 18/10/2018 | 38.807222 | 22.166667 | 250 |
| 49 | Belouchi | 18/10/2018 | 38.738333 | 22.277222 | 833 |
| 50 | Kefalovryso | 18/10/2018 | 38.698889 | 22.318333 | 178 |
| 51 | Paleomylos | 18/10/2018 | 38.889210 | 22.805630 | 333 |
| 52 | Ag. Dimitrios | 18/10/2018 | 38.908560 | 22.716330 | 250 |
| 53 | Megali Brisi | 18/10/2018 | 38.899120 | 22.487110 | 306 |
| 54 | Ag. Eleusa | 18/10/2018 | 38.629390 | 22.519860 | 154 |
| 55 | Kefalovryso Lilaia | 18/10/2018 | 38.640500 | 22.492170 | 106 |
| 56 | Ano Kefaloriso | 18/10/2018 | 38.630190 | 22.504490 | 331 |
| 57 | Polystavri | 19/10/2018 | 38.414210 | 22.068790 | 157 |
| 58 | Gouva | 19/10/2018 | 38.407080 | 21.917620 | 425 |
| 59 | Thermo | 19/10/2018 | 38.441460 | 21.980260 | 116 |
| 60 | Fleva | 19/10/2018 | 38.412092 | 22.059974 | 118 |
| 61 | Myloi | 19/10/2018 | 38.431590 | 22.397993 | 583 |
| 62 | Kirras p3 | 19/10/2018 | 38.422333 | 22.460022 | 1528 |
| 63 | Potamos | 19/10/2018 | 38.358692 | 22.602392 | 177 |
| 64 | Almyra Nera | 20/10/2018 | 38.629160 | 23.131710 | 756 |
| 65 | Rema | 20/10/2018 | 38.543240 | 23.282630 | 189 |
| 66 | Mylos Kokkosi | 20/10/2018 | 38.486100 | 23.478650 | 97 |
| 67 | Mavroneri est | 20/10/2018 | 38.525184 | 22.779784 | 972 |
| 68 | Charriton | 20/10/2018 | 38.499653 | 22.961018 | 2778 |
| 69 | Erkinna Krya | 20/10/2018 | 38.431756 | 22.875070 | 611 |
| 70 | Agios Ioannis | 20/10/2018 | 38.422283 | 22.459946 | 101 |
| 71 | Koprinitsi | 18/05/2019 | 37.271850 | 21.869910 | 71 |
| 72 | Ag. Floros | 18/05/2019 | 37.167900 | 22.026510 | 2600 |
| 73 | Pidima | 18/05/2019 | 37.137940 | 22.047270 | 260 |
| 74 | Logara | 18/05/2019 | 37.281310 | 22.246860 | 138 |
| 75 | Vivario | 18/05/2019 | 37.189670 | 22.375630 | 1900 |
| 76 | Methydrio | 18/05/2019 | 37.637770 | 22.176070 | 400 |
| 77 | Pyrgakio | 18/05/2019 | 37.634290 | 22.158970 | 250 |
| 78 | Sintzi | 19/05/2019 | 37.761540 | 22.341350 | 128 |
| 79 | Mpailtsakou | 19/05/2019 | 37.772990 | 22.221970 | 200 |
| 80 | Ladon | 19/05/2019 | 37.836820 | 22.182590 | 2000 |
| 81 | Planitero | 19/05/2019 | 37.933610 | 22.166210 | 3000 |
| 82 | Feneos | 19/05/2019 | 37.952610 | 22.334190 | 78 |
| 83 | Triza | 19/05/2019 | 37.869290 | 22.464250 | 187 |
| 84 | Moustos | 19/05/2019 | 37.385070 | 22.743670 | 80 |
| 85 | Lerna | 20/05/2019 | 37.552930 | 22.717050 | 2000 |
| 86 | Kefalari (Argos) | 20/05/2019 | 37.596100 | 22.688170 | 3333 |
| 87 | Kantia | 20/05/2019 | 37.519640 | 22.968420 | 800 |
| 88 | Epidavros (Ag. Nikolaos) | 20/05/2019 | 37.681200 | 23.150500 | 417 |
| 89 | Selontas | 20/05/2019 | 37.734130 | 23.117810 | 556 |
| 90 | Almyri | 20/05/2019 | 37.842290 | 23.016200 | 833 |
| 91 | Orea Eleni | 20/05/2019 | 37.870300 | 22.996880 | 556 |
| 92 | Mavrosoulava | 22/05/2019 | 38.258020 | 23.806620 | 1389* |
| 93 | Kaissarianis | 22/05/2019 | 37.965484 | 23.787821 | 4.44^ |
| 94 | Koroni | 27/02/2020 | 39.292960 | 20.542060 | 10000 |
| 95 | Skala | 27/02/2020 | 39.175580 | 20.764580 | 31000 |
| 96 | Agios Georgios | 27/02/2020 | 39.270160 | 20.851420 | 27000 |
| 97 | Vathy | 27/02/2020 | 39.308880 | 20.887830 | 18000 |
| 98 | Biros | 27/02/2020 | 39.432260 | 20.840240 | 800 |
| 99 | Likostomo | 27/02/2020 | 39.750340 | 20.773730 | 500 |
| 100 | Krya | 27/02/2020 | 39.719200 | 20.831390 | 200 |
| 101 | Perama | 27/02/2020 | 39.702000 | 20.857810 | 300 |
| 102 | Mana Nerou | 28/02/2020 | 40.048200 | 20.622100 | 245 |
| 103 | Bobos | 28/02/2020 | 40.004530 | 20.674250 | 250 |
| 104 | Nelles | 28/02/2020 | 39.982900 | 20.668690 | 160 |
| 105 | Doliana | 28/02/2020 | 39.894680 | 20.605910 | 1430 |
| 106 | Kalpaki | 28/02/2020 | 39.886450 | 20.624130 | 300 |
| 107 | Pigi Lithis | 29/02/2020 | 39.021960 | 22.954650 | 50 |
| 108 | Livari 2 | 29/02/2020 | 39.108960 | 22.933070 | 97 |
| 109 | Livari | 29/02/2020 | 39.111440 | 22.938190 | 20 |
| 110 | Tempi 2 | 29/02/2020 | 39.880820 | 22.586370 | 100 |
| 111 | Tempi | 29/02/2020 | 39.879450 | 22.585180 | 772 |
| 112 | Vryton | 07/03/2020 | 40.799736 | 21.918361 | 42 |
| 113 | Krystallopigi | 07/03/2020 | 39.491722 | 20.469770 | 150 |
| 114 | Petronikos | 20/07/2021 | 38.87061 | 21.17545 | 833 |
| 115 | Kryoneri | 20/07/2021 | 38.34475 | 21.59736 | 344 |
| 116 | Agios Stefanos | 21/07/2021 | 38.45546 | 23.61104 | 535 |
| 117 | Glyfa | 21/07/2021 | 38.54251 | 23.63823 | 300 |
| 118 | Kalamos Tsirloneri | 21/07/2021 | 38.40377 | 23.99841 | 139 |
| 119 | Almyropotamos | 21/07/2021 | 38.26894 | 24.16113 | 99 |
| 120 | Astakos | 22/07/2021 | 38.53502 | 21.10024 | 181 |
| 121 | Lambra | 22/07/2021 | 38.48393 | 21.23443 | 3889 |
| 122 | Anapsades | 23/07/2021 | 38.95836 | 21.62643 | 145 |
| 123 | Sotira | 23/07/2021 | 38.95952 | 21.6303 | 20 |
| 124 | Mega Rema | 23/07/2021 | 38.98303 | 21.68408 | 135 |
| 125 | Kefalovriso Karpenisi | 23/07/2021 | 38.89733 | 21.782 | 203 |
| 126 | Kefalovriso Thermo | 23/07/2021 | 38.5726 | 21.66633 | 194 |
| 127 | Kefalia Agia Sofias | 23/07/2021 | 38.5832 | 21.64904 | 184 |
| 128 | Prophitis Ilias | 23/07/2021 | 38.58570 | 21.61386 | 28 |
| 129 | Gorgogouvli | 24/07/2021 | 38.88204 | 20.99691 | 183 |
| 130 | Gouvo | 24/07/2021 | 38.93666 | 20.96777 | 139 |
| 131 | Vonitsa | 24/07/2021 | 38.9151 | 20.87498 | 278 |
| 132 | Monastiraki | 24/07/2021 | 38.85114 | 20.94386 | 157 |
| 133 | Korpi | 24/07/2021 | 38.86237 | 20.95509 | 28 |
| 134 | Nafpaktos | 25/07/2021 | 38.39344 | 21.83619 | 306 |
| 135 | Kallio | 25/07/2021 | 38.55769 | 22.17071 | 833 |
| 136 | Tempi 2 | 26/07/2021 | 39.87882 | 22.58695 | 100 |
| 137 | Ponte | 26/07/2021 | 39.89066 | 22.60649 | 50 |
| 138 | Dion | 26/07/2021 | 40.1736 | 22.49127 | 200 |
| 139 | Barouka | 28/07/2021 | 40.42120 | 20.81678 | 175 |
| 140 | Ano Arenes | 28/07/2021 | 40.34093 | 20.92366 | 289 |
| 141 | Kato Arenes | 28/07/2021 | 40.29520 | 20.95105 | 710 |
| 142 | Agios Fanourios | 29/07/2021 | 39.99811 | 20.55454 | 120 |
| 143 | Glavas | 29/07/2021 | 39.99186 | 20.54221 | 965 |
| 144 | Plataria | 03/10/2022 | 39.44032 | 20.27727 | 200 |
| 145 | Foiniki | 03/10/2022 | 39.61465 | 20.32675 | 200 |
| 146 | Xino Nero | 10/10/2022 | 40.69085 | 21.6215 | 36 |
| 147 | Anthochori | 10/10/2022 | 39.7363 | 21.12717 | 200 |
| 148 | Perama | 10/10/2022 | 39.70198 | 20.85812 | 300 |
| 149 | Vella | 11/10/2022 | 39.86604 | 20.62432 | 500 |
| 150 | Kastri | 11/10/2022 | 39.85682 | 20.614 | 200 |
| 151 | Zaravina Limni | 11/10/2022 | 39.90268 | 20.513 | 220 |
| 152 | Rogozi | 11/10/2022 | 39.91661 | 20.41189 | 700 |
| 153 | Arapi | 11/10/2022 | 39.96751 | 20.66476 | 540 |
| 154 | Voidomatis | 11/10/2022 | 39.94875 | 20.71385 | 1000 |
| 155 | Lista | 12/10/2022 | 39.73182 | 20.48038 | 1700 |
| 156 | Gerolimitsaina | 12/10/2022 | 39.73734 | 20.59214 | 100 |
| 157 | Kabi | 12/10/2022 | 39.21811 | 20.90188 | 650 |
| 158 | Acheron | 13/10/2022 | 39.32879 | 20.62144 | 1500 |
| 159 | Chohla | 13/10/2022 | 39.21797 | 20.5658 | 2400 |
| 160 | Krioneri | 13/10/2022 | 39.45793 | 21.12387 | 30 |
| 161 | Makaria | 15/10/2022 | 38.15811 | 24.00212 | 500 |
| 162 | Dimitsana | 15/10/2022 | 37.58315 | 22.04741 | 51.4 |
| 163 | Kefalari Kakouraiika | 15/10/2022 | 37.58244 | 21.91908 | 50 |
| 164 | Agia Mama | 16/10/2022 | 37.16643 | 22.30795 | 36.9 |
| 165 | Tripi | 16/10/2022 | 37.09362 | 22.348 | 41.7 |
| 166 | Sotiros | 16/10/2022 | 36.98629 | 22.40815 | 196.4 |
| 167 | Kefalovryso Lianos | 17/10/2022 | 37.37684 | 22.31037 | 41.7 |
| 168 | Maggani | 17/10/2022 | 37.79661 | 22.12101 | 47.2 |
| 169 | Mati | 17/10/2022 | 37.8214 | 22.10626 | 47.2 |
| 170 | Kertezi | 17/10/2022 | 37.97772 | 21.99292 | 65 |
| 171 | Tria Piagadia | 17/10/2022 | 38.0376 | 22.09863 | 50 |
| 172 | Mpala | 17/10/2022 | 38.25498 | 21.80866 | 83.3 |

***** total pumping rate of the well field; ^ pumping rate of a single well

**S1. Karst hydrosystems in Greece**

The karst systems in Greece were developed during the Alpine orogeny (Jurassic – Miocene). The karstification process of the carbonate formations was the result of lateral movements in the upper Eocene, intense tectonism at the end of the upper Miocene, and eustatic movements of the Mediterranean Sea during glacial and interglacial periods (Katsanou, 2018). According to Papadopoulou-Vrinioti (1993), the Hellenic karst systems are distinguished into three groups: (i) “paleokarst”, developed during warm-wet climates (upper Cretaceous; upper-lower Miocene) and is considered as fossilized; (ii) “younger karst”, developed during the interglacial periods (Pleistocene-Holocene); (iii) “recent karst”, developed under the current climatic conditions. Furthermore, the karst features are determined by three factors: (i) Messinian salinity crisis caused karstification in great depth below the present sea level; (ii) Quaternary cold periods caused weathering of the epikarst; (iii) post-Miocene tectonics created horst and graben structures with the filling of large basins by thick sedimentary sequences (Bakalowicz, 2015). The formation of karstic systems below the present sea level is generally attributed to tectonic movements and/or sea level drops during glacial periods (Fleury et al., 2007).

The main Hellenic aquifers are hosted in clastic Quaternary and Neogene deposits and in carbonate rocks, in which groundwater is abstracted via several wells and boreholes (about 300,000 for the whole Greek territory - Daskalaki & Voudouris, 2008). Karst aquifers are developed both in carbonate sedimentary rocks, limestones and dolomites (Triassic – Cretaceous), and in carbonate metamorphic rocks, Paleozoic – Mesozoic marbles (Voudouris & Kazakis, 2018). Furthermore, some karst hydrosystems are developed in evaporitic formation; high-sulfate karst springs are found in western Greece within the Ionian zone, which can be associated with gypsum dissolution (Rigakis and Karakitsios, 1998; Nikolaou et al., 2011; Katsanou et al., 2017). Carbonate formations cover about 35% of the country and are mainly located in Western, Central, and Southern Greece (Daskalaki & Voudouris, 2008). Many of them (55% of karst aquifers) reach coastal areas and often extend below sea level. Examples can be found in Central Greece (South Parnassos and Ghiona aquifers), in Eastern Peloponnese (Tripolis and East Argolis aquifers), and in Crete (the famous Almyros spring) (Kallioras & Marinos, 2015; Voudouris & Kazakis, 2018). The Mediterranean area is known for its widespread presence of coastal and submarine karstic springs, which are estimated to represent about 90% of the whole world (Bakalowicz, 2015).

Most of the country’s karst aquifers have a holokarst-type shape, characterized by well-developed karst features extended below base levels and high hydraulic conductivity; holokarst are very common in the limestones of the Ionian, Gavrovo-Tripolis, Parnassos-Giona, and Pelagonian zones that outcrop in Western and Central Greece and Crete Island.

The hydrogeological behaviour is controlled by intense tectonic deformation, which favours infiltration and karstification processes (Kallioras & Marinos, 2015). According to Voudouris & Kazakis, 2018, the hydraulic conductivity of karst aquifers ranges from 10^-2^ m s^-1^ to 10^-6^ m s^-1^, the transmissivity varies between 30 m^2^ h^-1^ and 300 m^2^ h^-1^, while the maximum groundwater velocity is up to 1000 m h^-1^.

**S2. Supplementary discussion on trace elements in the karst hydrosystems of Greece**

*S2.1. Strontium*

Strontium is a lithophile metallic element and a common trace element in groundwater. Because of a similar ionic ratio and charge to calcium, Sr behaves similarly in the environment and can replace it. Indeed, intake of high Sr quantities can have negative effects on human health, such as abnormal bone development in young people (ATSDR, 2004). Although the WHO has not set a maximum admissible concentration in drinking water, the USEPA has set a non-regulatory benchmark, a health-based screening level (HBSL), of 4000 µg L^-1^ to indicate a potential human-health concern (Norman et al., 2018). Strontium derives from several sources, both natural and anthropogenic. It derives from weathering of some Sr-bearing minerals, as well as carbonates, evaporites, and granites. The main Sr-bearing minerals are celestite (SrSO_4_) and strontianite (SrCO_3_). In addition, strontium may have a marine source due to seawater-groundwater mixing. Moreover, Sr can be associated with industrial waste, mining, or landfill leachate (Musgrove, 2021 and references therein).

In the present study, Strontium concentration in karst springs ranges between 41.1 µg L^-1^ and 8200 µg L^-1^ (Fig.2b). The highest concentrations are found in some karst springs located in coastal areas or in Epirus, where evaporite outcrops are present (Fig.SM3a). Strontium of the former springs derives from the mixing between karst groundwater and seawater, whilst the strontium concentration of Epirus springs derives from the dissolution of celestite and strontianite minerals hosted in evaporite formations. While both water types show Sr/SO_4_ ratios higher than that of seawater, the waters that interacted with evaporites show generally the highest ratios. In Epirus, Triassic gypsum-rich evaporites include celestite minerals, whose precipitation is favoured by the dissolution of Ca-sulfate. The samples reach even saturation in celestite (Fig.SM3b). Strontium concentrations in Greek evaporites vary from 1100 ppm to 64,270 ppm (Badouna et al., 2021), being sometimes very high and, therefore, justifying the Strontium values found in the sampled karst springs. Some of the sampled waters exceed the above-mentioned USEPA limit, but these are all saline waters anyway not suitable for human consumption.

*S2.2. Chromium and Nickel*

Chromium appears in the natural environment in trivalent [Cr(III)] and hexavalent [Cr(VI)] oxidation states. Cr(III) is considered an essential element for human physiology; on the contrary, Cr(VI) is highly soluble in water and is considered an extremely toxic form (Apollaro et al., 2019). Nickel is a mobile metallic element and in natural water exists in the oxidation states, Ni(II), Ni(III), and Ni(IV), and, like chromium, is considered a toxic element (Das et al., 2019).

Chromium and Nickel pollution may derive from both natural and anthropogenic sources. A natural source is the weathering of Cr-Ni-rich minerals in ultramafic and ophiolitic complexes (Salminen, 2005; De Vos and Tarvainen, 2006), whilst anthropogenic pollution derives from extensive use of Cr-based chemicals in industrial activities (Reimann and de Caritat, 1998).

In Greece, natural Cr and Ni contaminations are observed in areas where the aquifer is hosted in the ophiolitic belts of Pindos and Vardar (Li Vigni et al, 2021; Vasileiou et al., 2019). High levels of Cr(VI) were observed in the drinking waters of Central Macedonia, Thessaly, Attica, Lesvos, and Rhodes Islands (Kaprara et al., 2015).

In the present study, only total chromium was measured, but Cr(VI) is likely the dominant form, due to its great mobility with respect to Cr(III). In the analysed karst springs (Fig.2b), both Cr (up to 6.58 µg L^-1^) and Ni (up to 6.91 µg L^-1^) show values far from the limits set by the European Council (50 µg L^-1^ for Cr and 20 µg L^-1^ for Ni). The Hellenic karst water, like most freshwater worldwide (Reimann & de Caritat, 1998), shows low Cr and Ni concentrations, not evidencing contaminations either of anthropogenic or natural origin. Although in Greece many studies evidenced Cr and Ni contaminations deriving from the leaching of widespread ophiolitic sequences in the country (Dermatas et al., 2015; Economou-Eliopoulos et al., 2017; Tziritis et al., 2012) or lateritic horizons (Tziritis, 2014), no similar impact has been evidenced on the presently studied karstic waters.

*S2.3. Lead*

Lead is a toxic heavy metal element, forming minerals like galena (PbS) and cerussite (PbCO_3_). In groundwaters, Pb derives naturally from the leaching of minerals associated with ore deposits. Among anthropogenic sources, mining activities, leaded gasoline, batteries, paints, and water-plumbing systems are considered the primary lead pollutants for groundwater. According to WHO (2022), lead was recognised as one of the ten chemicals of major concern to public health, due to its widespread use. The major risk is the neurotoxic effect on children (WHO, 2016). The 98/83 European Directive set the limit for drinking water to 10 µg L^-1^, but the new directive (2020/2184/EC) lowered the limit to 5 µg L^-1^, which must be reached by 2036.

In Greece, elevated Pb concentrations in groundwater (up to 2700 µg L^-1^ in Eastern Central Greece) were observed in the whole country, except in Epirus and Central Macedonia (Dokou et al., 2015). In the karst springs collected for this study, Pb concentrations reached up to 2.36 µg L^-1^, not exceeding the European Directive. The highest values (> 0.1 µg L^-1^) were found in the karst springs of Euboea Island and West Central Greece. In the same areas, Dokou et al. (2015), who did not consider only karstic groundwater, estimated Pb concentration ranges of 10-50 µg L^-1^ and 7.5-10 µg L^-1^, respectively, higher values than those found in this study.

**Supplementary references**

Apollaro C., Fuoco I., Brozzo G., De Rosa R. Release and fate of Cr(VI) in the ophiolitic aquifers of Italy: the role of Fe(III) as a potential oxidant of Cr(III) supported by reaction path modelling. *Sci. Total Environ*. **660**, 1459-1471 (2019).

ATSDR. Toxicological profile for Strontium. Agency for Toxic Substances and Disease Registry Atlanta, GA: U.S. Department of Health and Human Services, Public Health Service (2004).

Badouna, I. *et al*. Mineralogical and geochemical properties of greek evaporites, associated with their prospects of industrial use. *Mater. Proc*. **5**, 3 (2021). https://doi.org/10.3390/materproc2021005003

Bakalowicz M. Karst and karst groundwater resources in the Mediterranean. *Environ. Earth Sci*. **74**, 5-14 (2015).

Das, K.J. *et al*. Primary concept of nickel toxicity – an overview. *J. Basic Clinic Physiol. Pharmacol*. **30**, 141-152 (2019). https://doi.org/10.1515/jbcpp-2017-0171

Daskalaki P., Voudouris K. Groundwater quality of porous aquifers in Greece: a synoptic review. *Environ. Geol*. **54**, 505-513 (2008).

Dermatas, D. *et al*. Origin and concentration profile of chromium in a Greek aquifer. *J. Hazard. Mater*. **281**, 35-46 (2015).

De Vos, W., Tarvainen, T. (Chief eds.) *Geochemical Atlas of Europe. Part 2* - Interpretation of Geochemical Maps, Additional Tables, Figures, Maps, and Related Publications. Geological Survey of Finland, ISBN: 951-690-960-4 (2006).

Dokou, Z., Kourgialas, N.N. & Karatzas, G.P. Assessing groundwater quality in Greece based on spatial and temporal analysis. *Environ. Monit. Assess*. **187**, 774 (2015). https://doi.org/10.1007/s10661-015-4998-0

Economou-Eliopoulos M., Megremi I., Vasilatos Ch., Frei R., Mpourodimos I. Geochemical constraints on the sources of Cr(VI) contamination in waters of Messapia (Central Evia) Basin. *Appl. Geochem*. **84**, 13-25 (2017).

Fleury, P., Bakalowicz, M., de Marsily, G. Submarine springs and coastal karst aquifers: a review. *J. Hydrol*. **339**, 79–92 (2007).

Kallioras, A., Marinos, P. Water resources assessment and management of karst aquifer systems in Greece. *Environ. Earth Sci*. **74**, 83-100 (2015).

Kaprara, E. *et al*. Occurrence of Cr(VI) in drinking water of Greece and relation to the geological background. *J. Hazard. Mater*. **281**, 2-11 (2015).

Katsanou, K. Hellenic Karst Aquifers Vulnerability Approach Using Factor Analysis: The Example of the Louros Karst Aquifers. *Geosciences* **8**, 417 (2018).

Katsanou, K., Lambrakis, N., D’Alessandro, W., Siavalas, G. Chemical parameters as natural tracers in hydrogeology: A case study of Louros Karst System, Greece. *Hydrogeol. J*. **25**, 487-499 (2017). DOI: 10.1007/s10040-016-1492-x

Li Vigni, L., Daskalopoulou, K., Calabrese, S., Parello, F., D’Alessandro, W., Geochemical characterization of the alkaline and hyperalkaline groundwater in the Othrys Ophiolite Massif, central Greece. *It. J. Geosci*. **140**, 42-56 (2021).

Musgrove, M. The occurrence and distribution of strontium in U.S. groundwater. *Appl. Geochem*. **126**, 104867 (2021).

Nikolaou, E., Pavlidou, S., Katsanou, K. Aquifer systems of Epirus, Greece: An overview. In: *Advances in the Research of Aquatic Environment* (Lambrakis, N., Stournaras, G., Katsanou, K. eds.) Springer, Berlin, Germany, vol. 1, 425-433 (2011).

Norman, J.E., Toccalino, P.L., Morman, S.A. Health-based screening levels for evaluating water-quality data. US Geological Survey web page, <https://water.usgs.gov/nawqa/HBSL> (accessed 18/03/2023) (2018). DOI 10. 5066/F71C1TWP.

Papadopoulou-Vrinioti, K. Hellenic karst and Environment. In: *Proceedings of the 3^rd^ Pan-Hellenic Geographical Conference*, Athens, Greece, 1-3 April 1999, pp. 290-299 (1999).

Piperopoulou, Ch., Stathaki, S., Drosos, G., Panou-Dimanti, Ou., Vottea Ei., Business plan for the development of a business park in the Greek territory, in accordance with article 42 par. 3 of Law 3982/2011. Summary/Conclusions n.n.: Support for Development and Competitiveness. General Secretariat of Industry, 17-19 (2018)

Reimann, C, et al. *Environmental Geochemical Atlas of the Central Barents Region*. ISBN 82-7385-176-1. NGU-GTK-CKE Special Publication, Geological Survey of Norway, Trondheim, Norway, 745 pp. (1998)

Rigakis, N., Karakitsios, V. The source rock horizons of the Ionian Basin (NW Greece). *Mar. Petrol. Geol*. **15**, 593-617 (1998).

Salminen, R. (Chief Ed.) *FOREGS Geochemical Atlas of Europe, Part 1: Background Information, Methodology and Maps*. Geological Survey of Finland, ISBN: 951-690-921-3 (2005).

Tsirambides A., Filippidis A. Energy mineral resources of Greece. *J. Environ. Sci Engin. B* **2012/6** 709-719 (2012). DOI:10.17265/2162-5263/2012.06.001

Tziritis, E. Environmental monitoring of Micro Prespa Lake basin (Western Macedonia, Greece). Hydrogeochemical characteristics of water resources and quality trends. *Environ. Monit. Assess*. **186**, 4553-4568 (2014).

Tziritis, E., Kelepertzis, E., Korres, G., Perivolaris, D., Repani, S. Hexavalent chromium contamination in groundwaters of Thiva Basin, Central Greece. *Bull. Environ. Contam. Toxicol*. **89**, 1073-1077 (2012).

Vasileiou, E., Papazotos, P., Dimitrakopoulos, D., Perraki, M. Expounding the origin of chromium in groundwater of the Sarigkiol Basin, Western Macedonia, Greece: A cohesive statistical approach and hydrochemical study. *Environ. Monit. Assess*. **191**, 509 (2019).

Voudouris, K. Status and codification of karst aquifer systems in Greece. *Bull. Geol. Soc. Greece* **57**, 23-51 (2019).

Voudouris, K., Kazakis, N. General characteristics and classification of karst aquifers in Greece. *Rev. Bulgar. Geol. Soc*. **79**, 159-160 (2018).

WHO. *Lead in drinking water. Background document for development of WHO guidelines for drinking water quality*. Geneva, World Health Organization (2016).

WHO. *Lead in drinking water. Health risks, monitoring and corrective actions*. Technical brief. Geneva, World Health Organization (2022).

**Figure SM1.** Geographic distribution of collected Hellenic karst springs associated to the corresponding identification number (Table SM1). The letter of each map refers to Fig. 1 in the main text. Karst hydrosystems from Voudouris (2019). Basemap by *ESRI* maps.


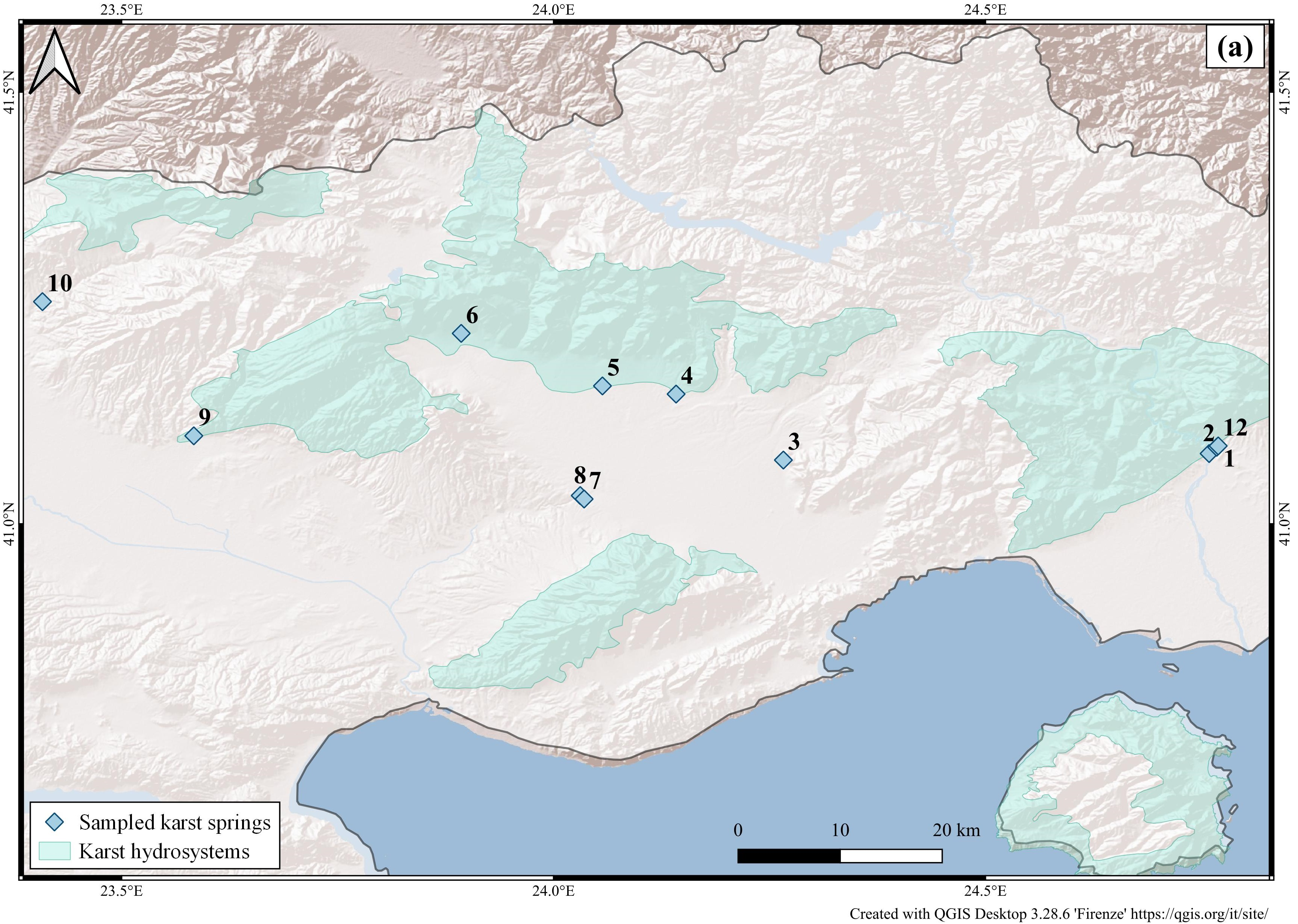


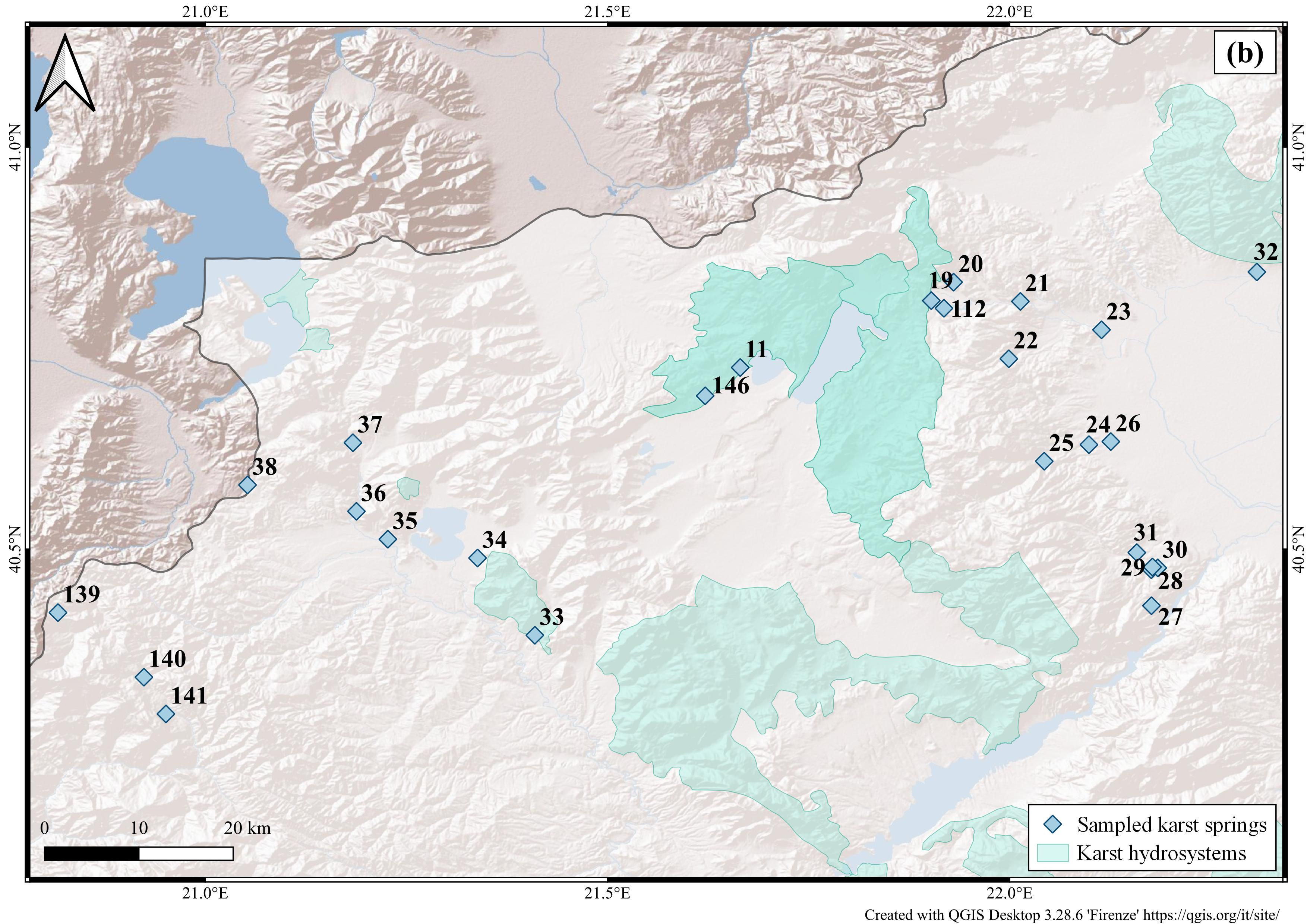


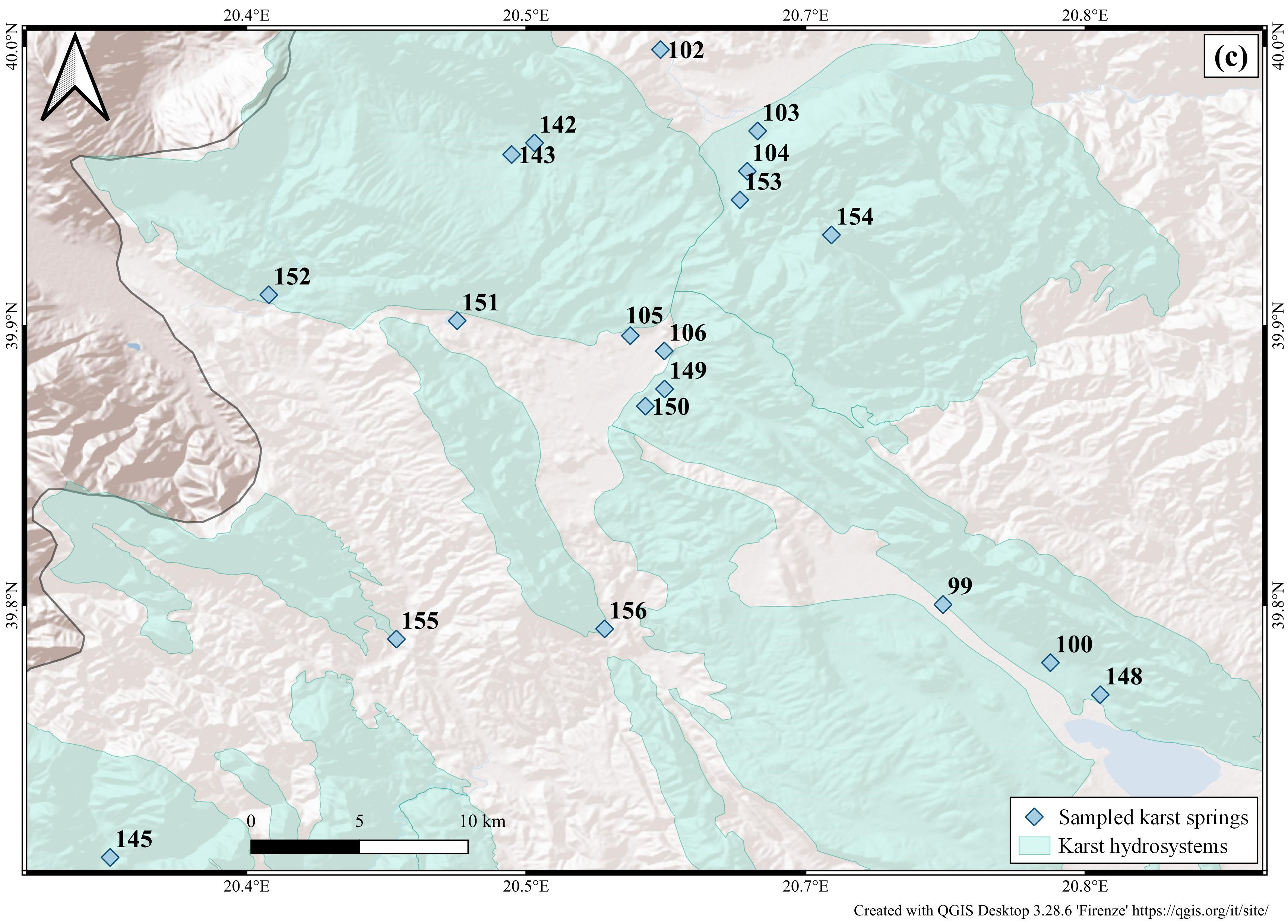


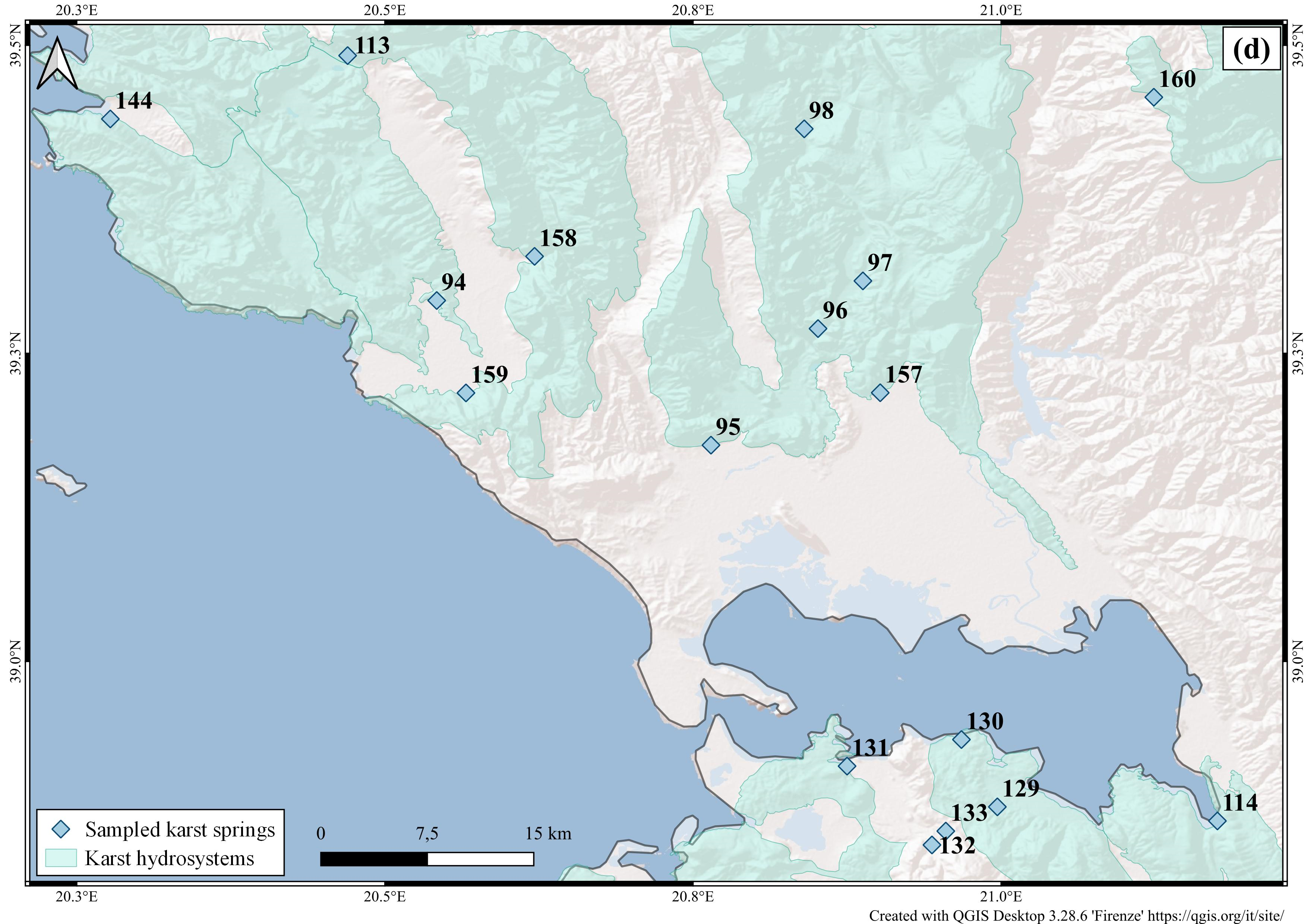


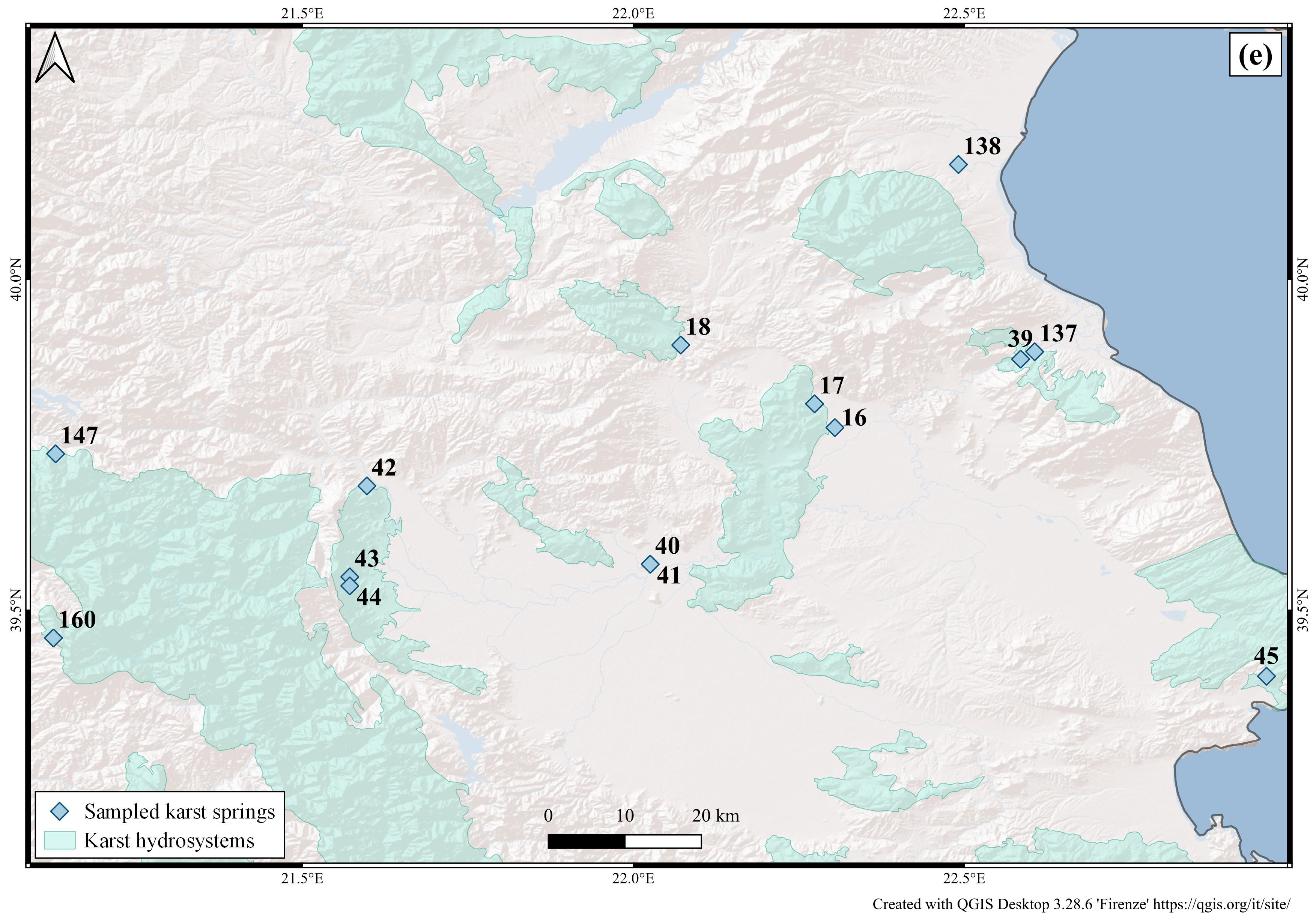


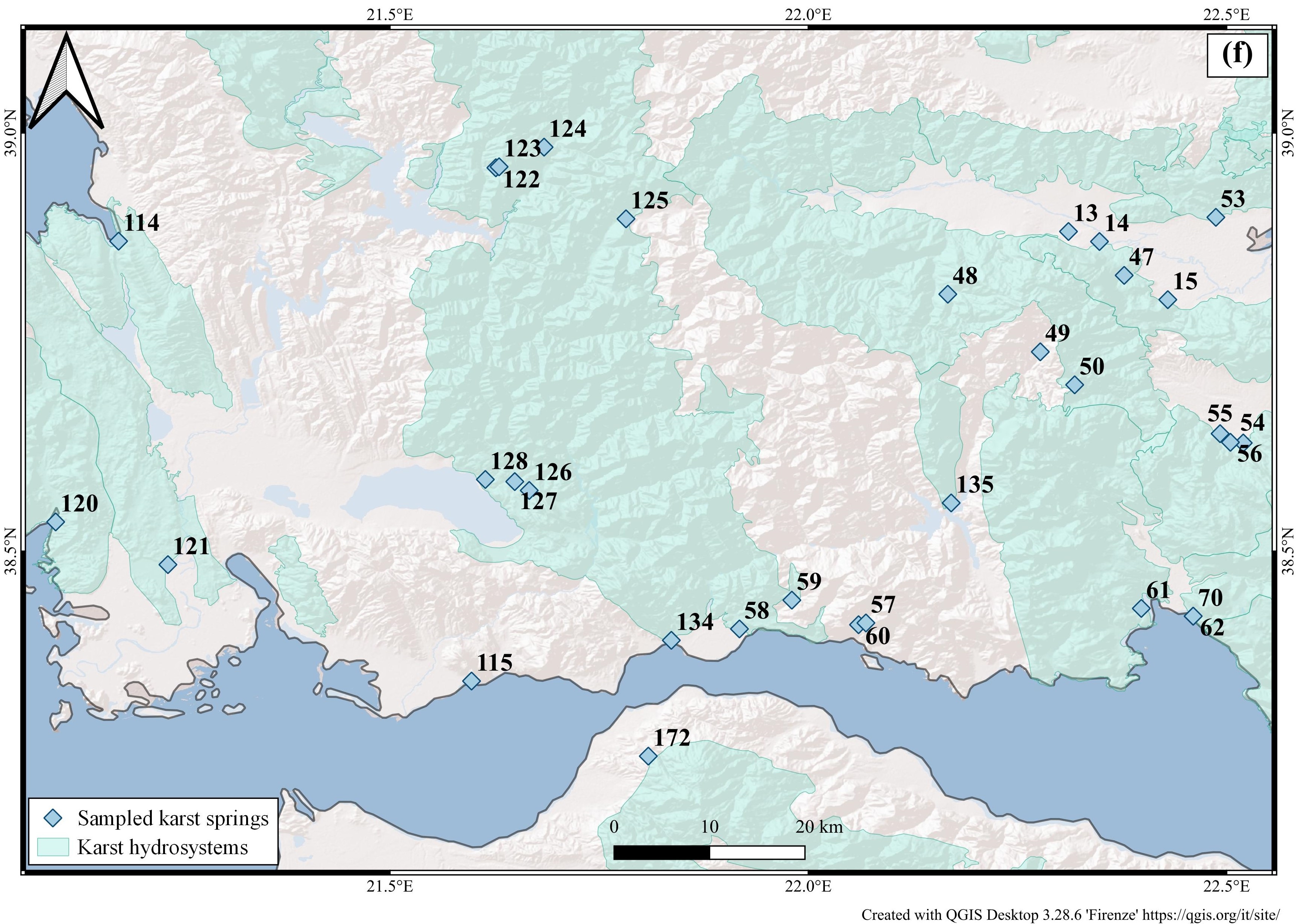


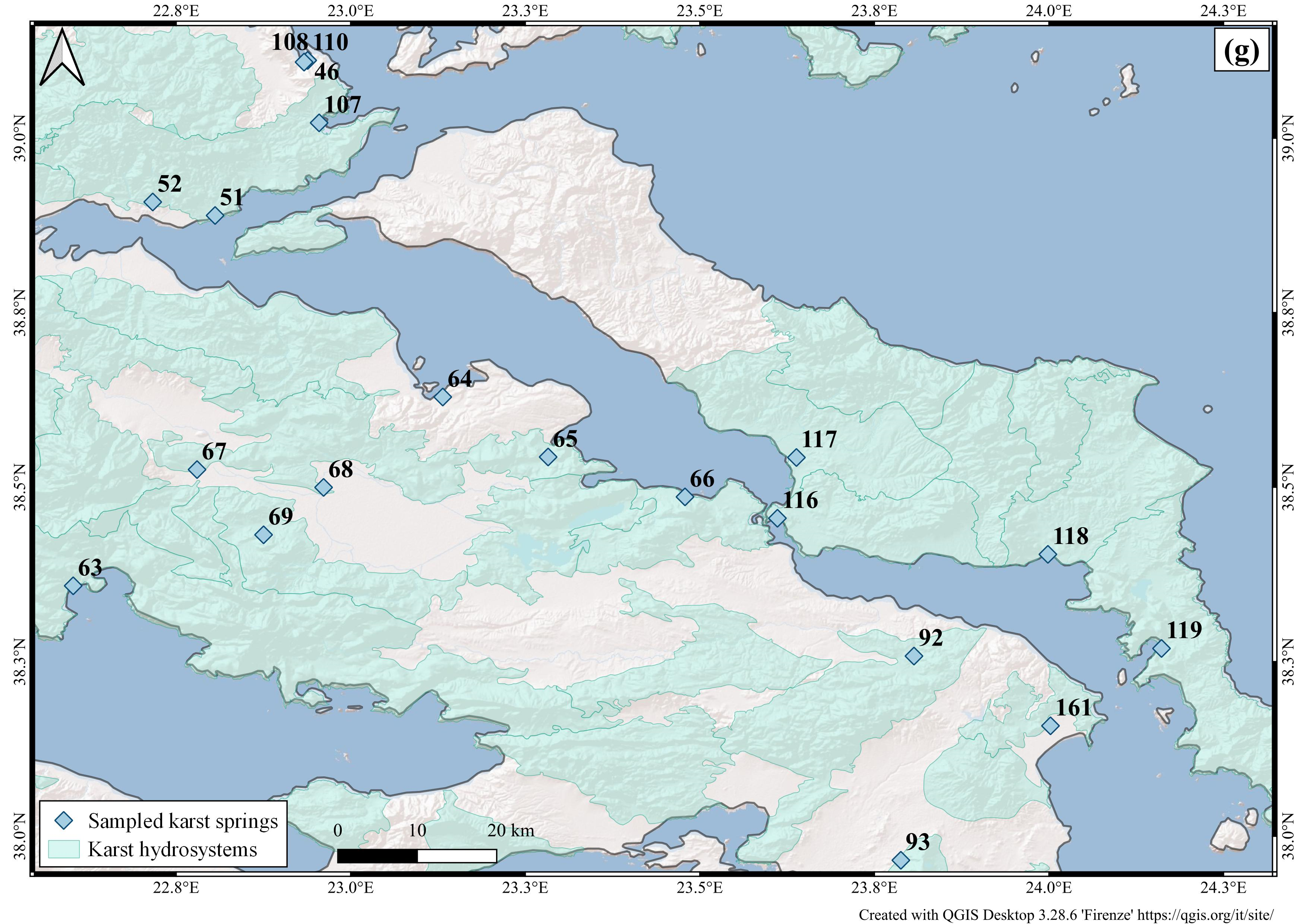


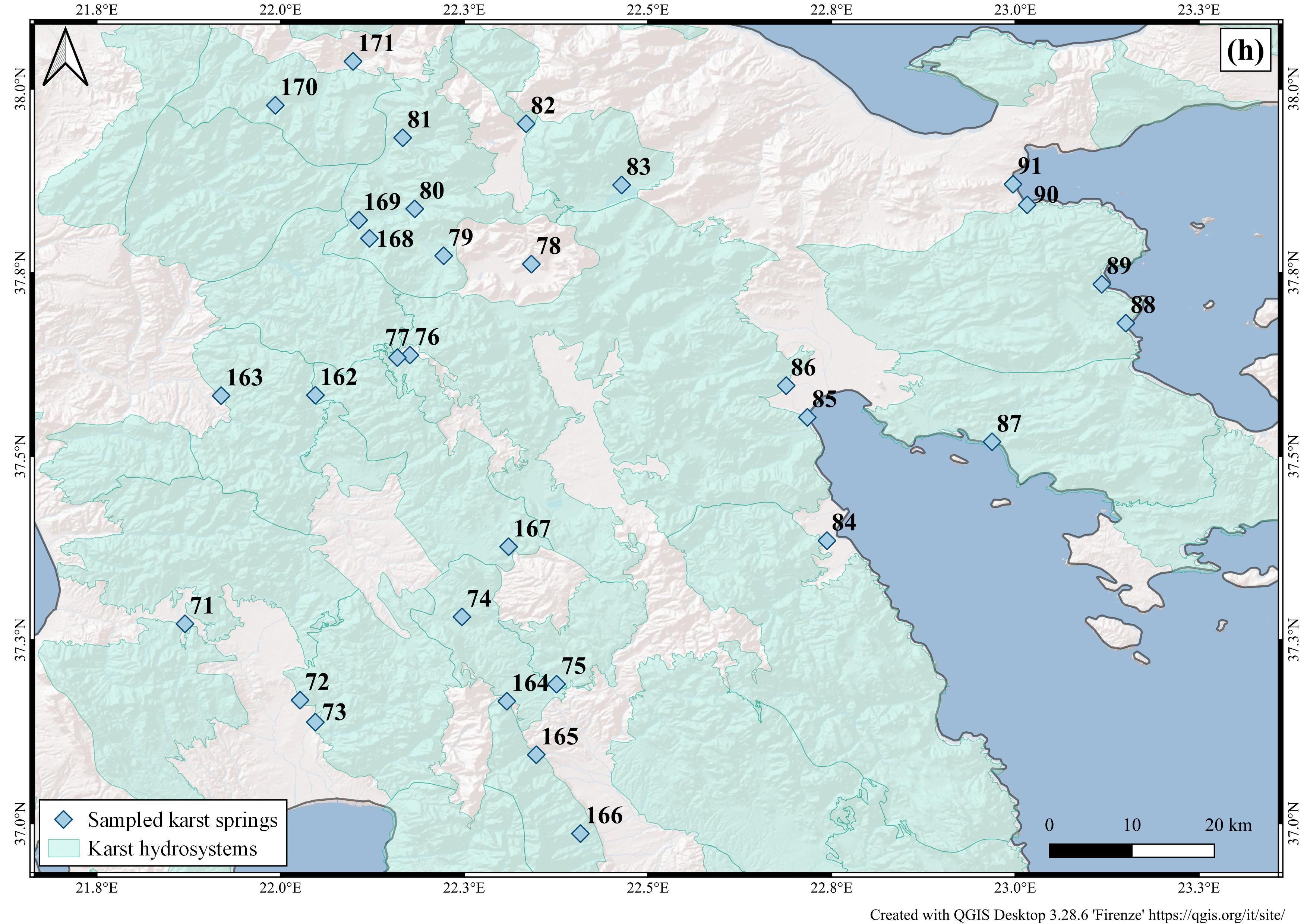


**Figure SM2**. Geographical distribution of sulfate karst springs. Karst hydrosystems from Voudouris (2019). Basemap by *ESRI* maps.


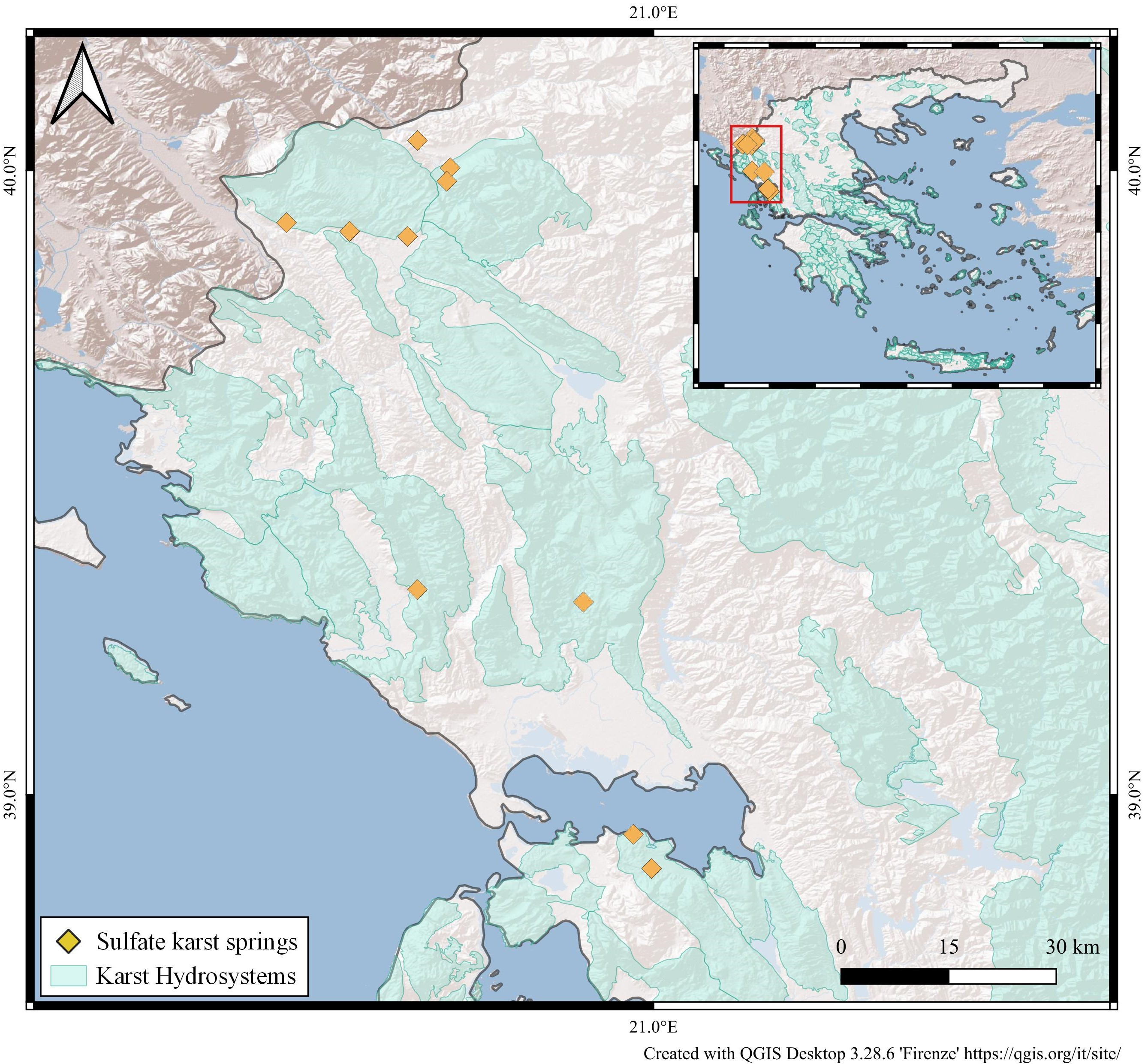


**Figure SM3**. Binary correlation plots of sulfate karst springs. (a) Sr versus Cl^-^; (b) saturation index of celestite versus SO_4_^2-^ (the shaded stripe is the ±0.5 range of values in which the waters are considered at saturation with respect to the solid phase).

**Figure SM4** – Distribution map of chloride concentrations in the karstic waters.


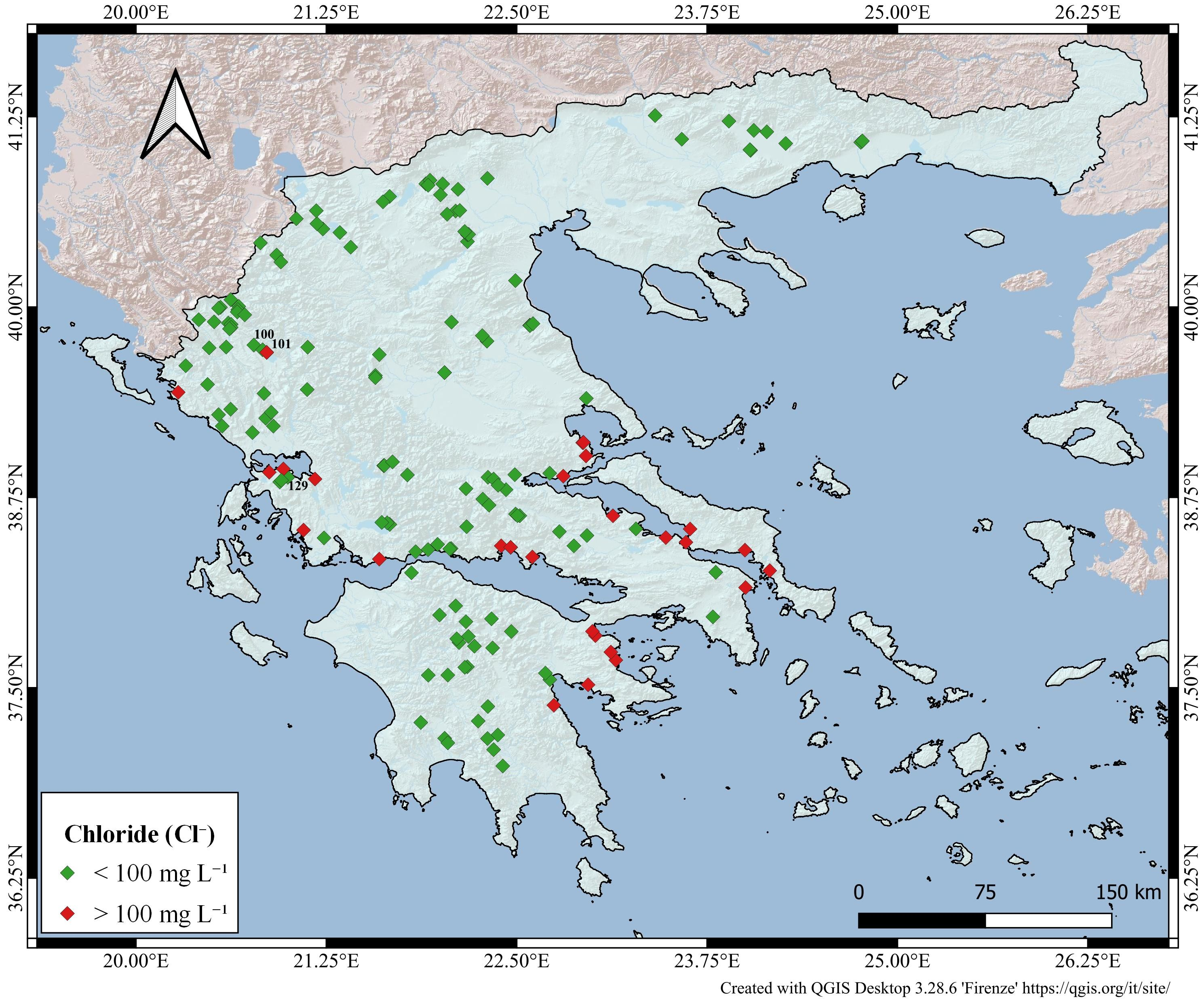


**Figure SM5** – Distribution map of nitrate concentrations in the karstic waters.


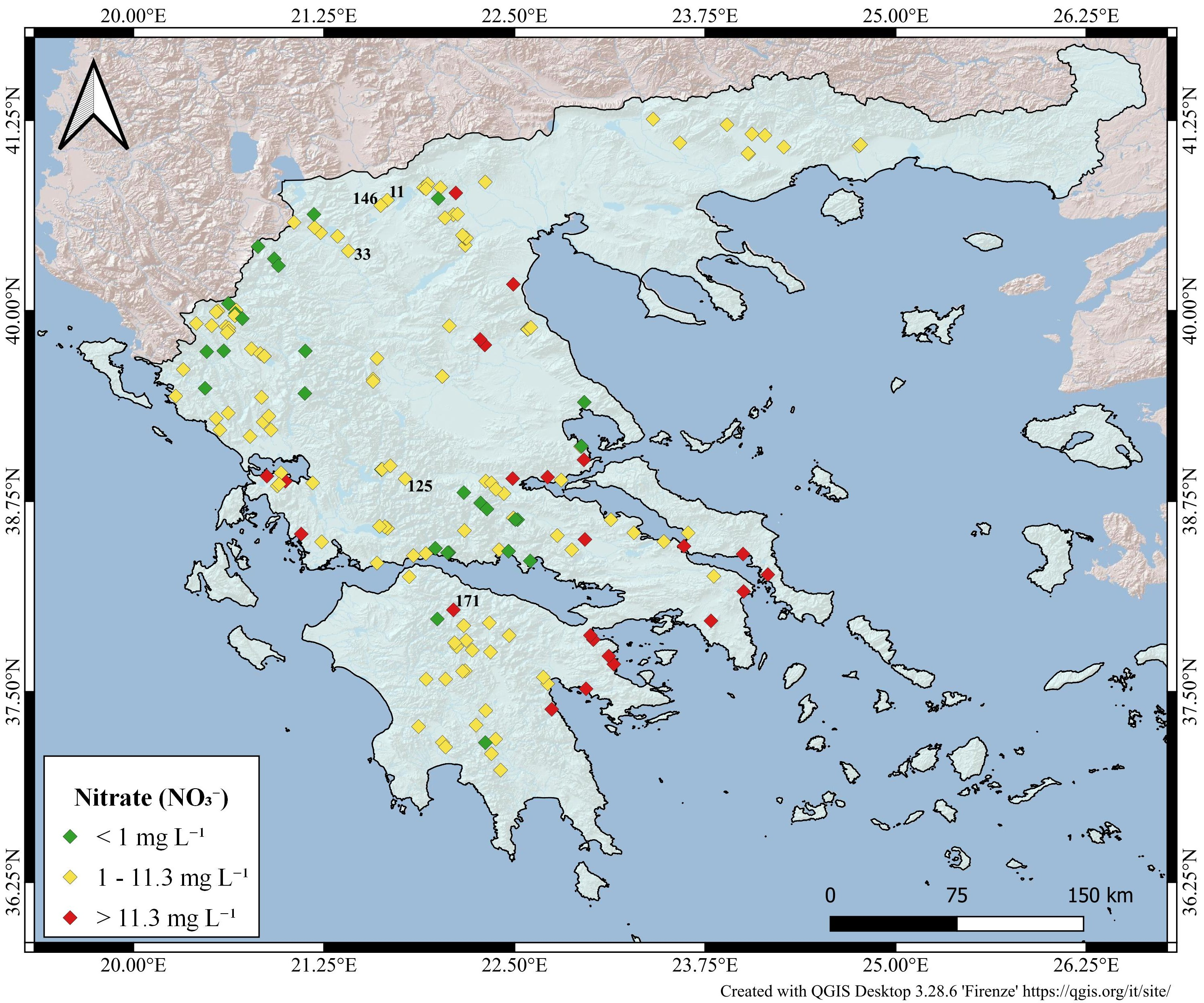


**Figure SM6** – Map of the main agricultural areas and of the population densities. Data from CORINE Land Cover 2018 (https://land.copernicus.eu/pan-european/corine-land-cover/clc2018).


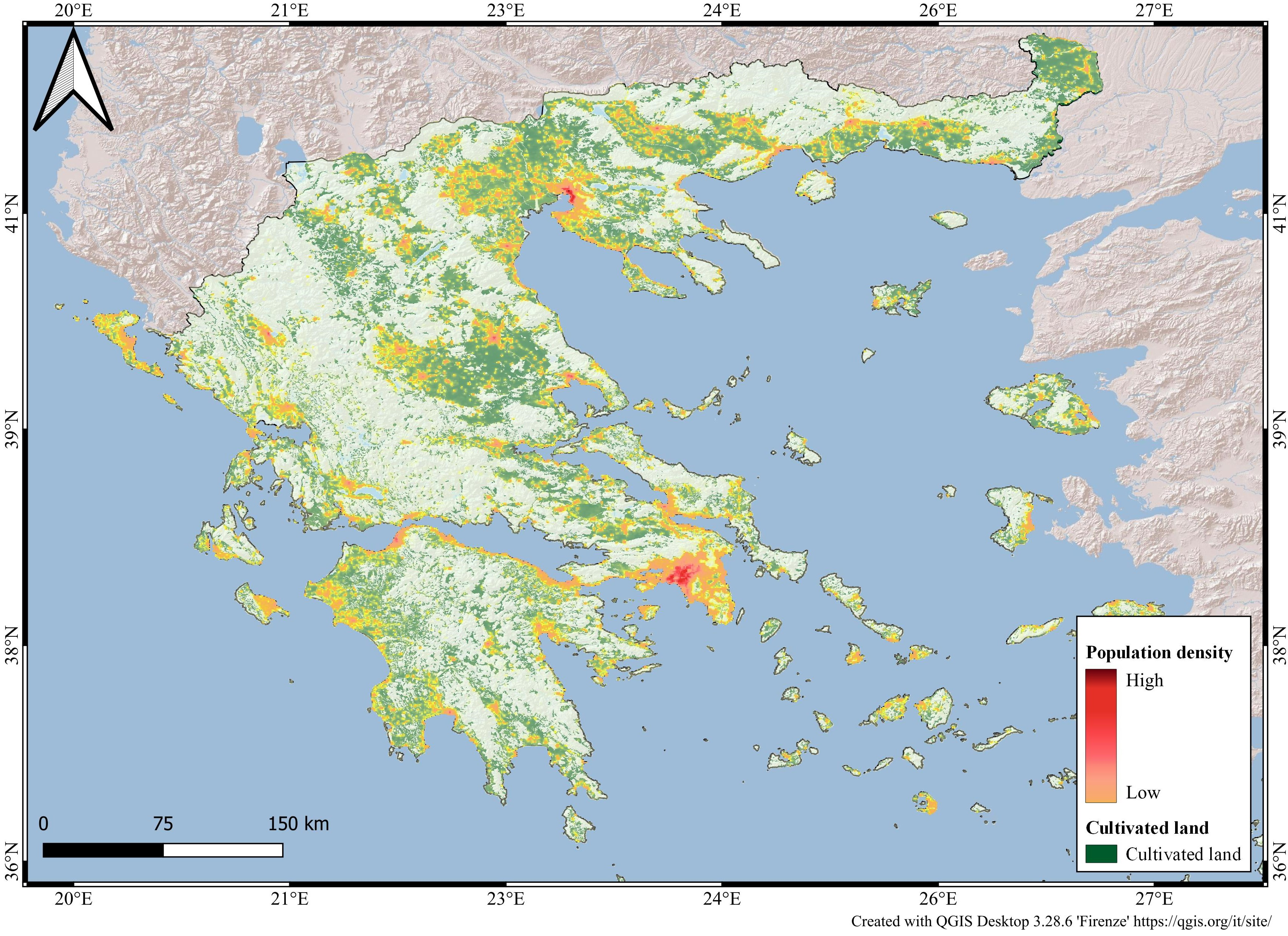


**Figure SM7** – Distribution map of arsenic concentrations in the karstic waters.


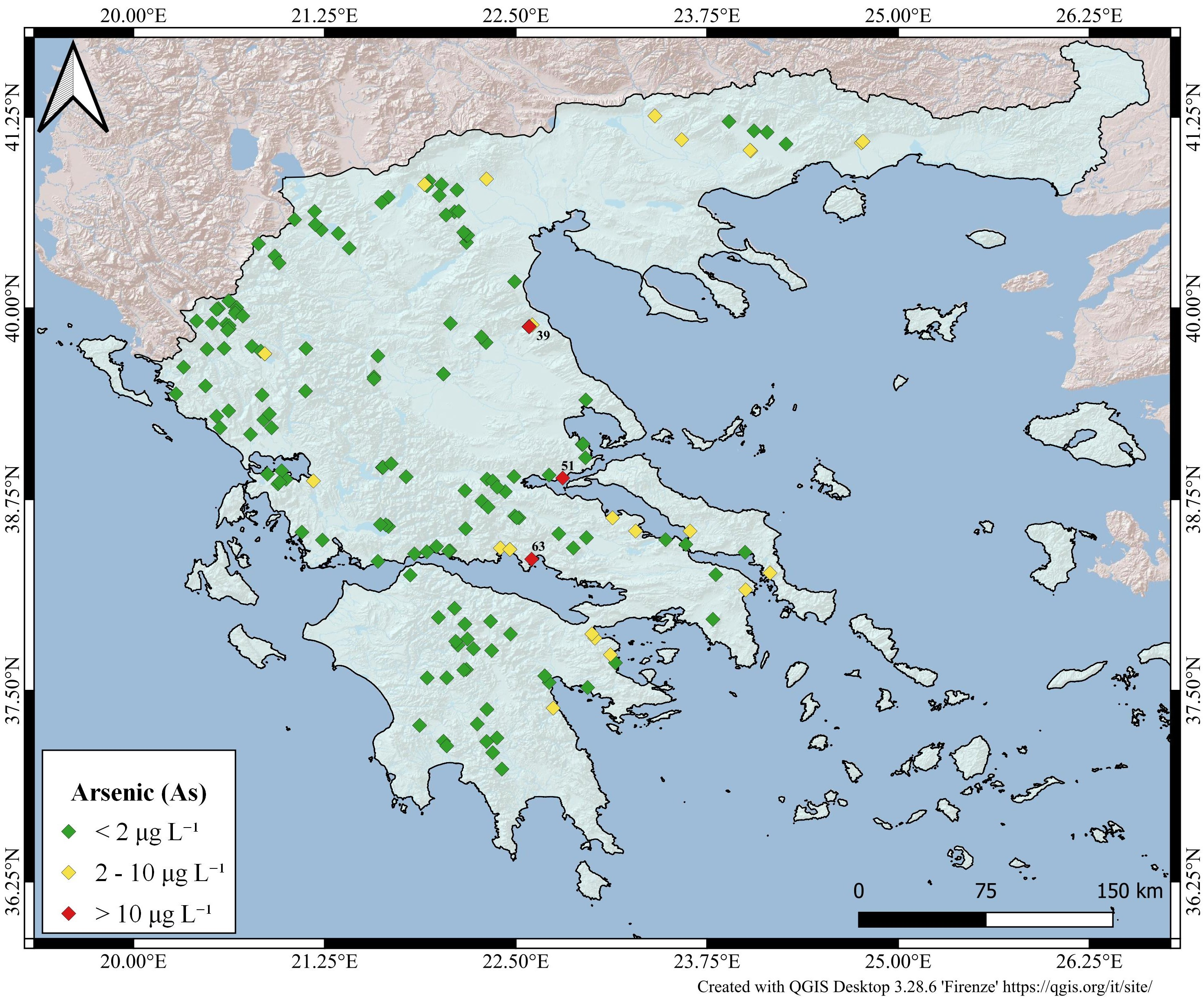


**Figure SM8** – Map of the main industrial areas (Piperopoulou et al., 2018) and of the main mineralizations (Tsirambides and Filippidis, 2012) in Greece.


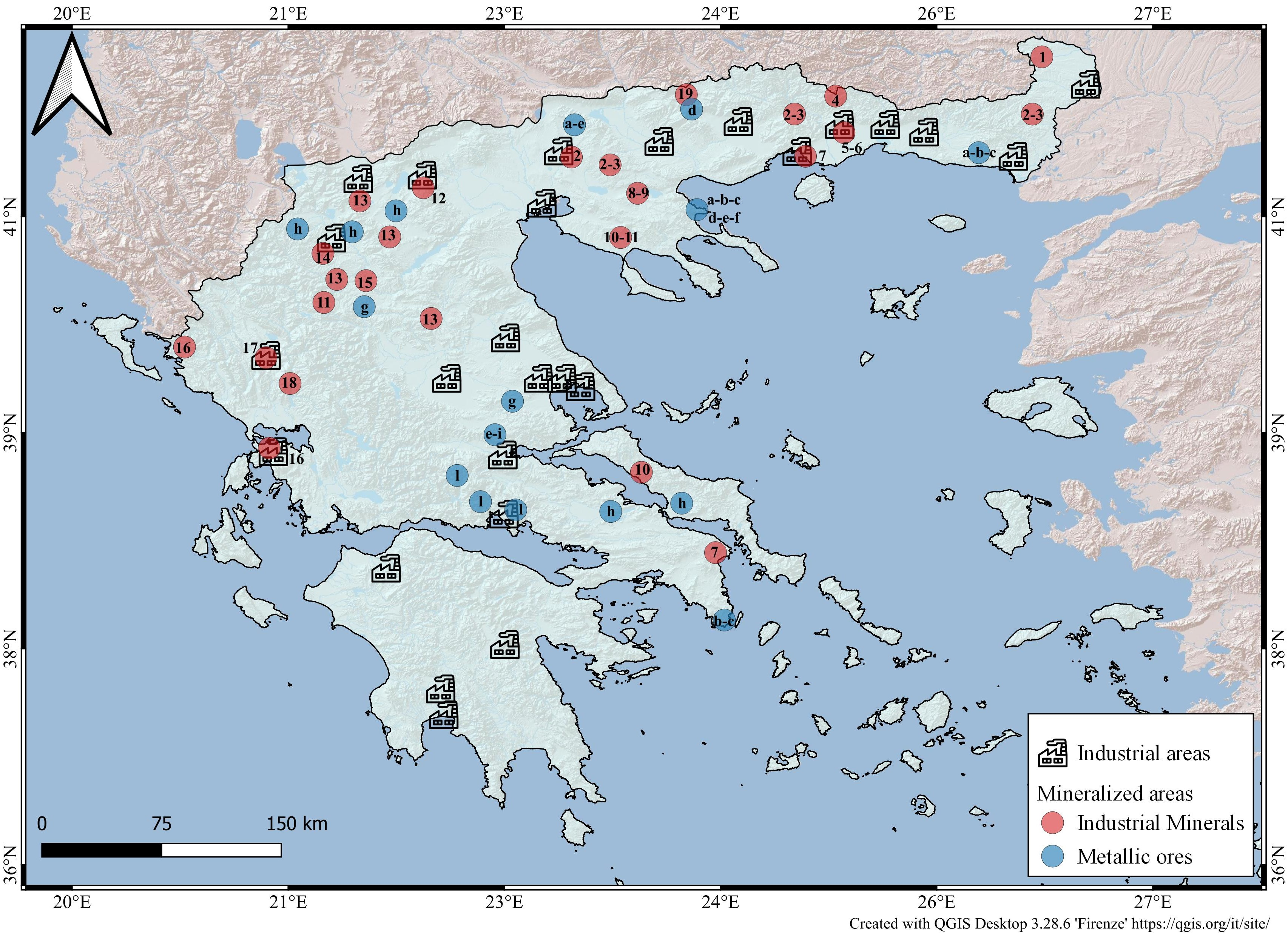


1 = Zeolite; 2 = Quartz; 3 = Feldspars; 4 = Graphite, 5 = Wollastonite; 6 = Garnet; 7 = White carbonates; 8 = Talc; 9 = Vermiculite; 10 = Magnesite; 11 = Olivine; 12 = Pozzolan; 13 = Diatomite; 14 = Attapulgite; 15 = Huntite; 16 = Gypsum; 17 = Phosporites; 18 = Halite; 19 = Kaolin.

a = Gold; b = Lead; c = Zinc; d = Manganese; e = Copper; f = Silver; g = Chromium; h = Nickel; i = Pyrite; l = Bauxite
